# Photochemical and thermal intramolecular 1,3-dipolar cycloaddition reactions of new *o*-stilbene-methylene-3-sydnones and their synthesis

**DOI:** 10.3762/bjoc.7.196

**Published:** 2011-12-13

**Authors:** Kristina Butković, Željko Marinić, Krešimir Molčanov, Biserka Kojić-Prodić, Marija Šindler-Kulyk

**Affiliations:** 1 Department of Organic Chemistry, Faculty of Chemical Engineering and Technology, University of Zagreb, Marulićev trg 19, 10 000 Zagreb, Croatia; 2 present address: Galapagos istraživački centar, Prilaz baruna Filipovića 29, 10 000 Zagreb, Croatia; 3 Center for NMR, Rudjer Bošković Institute, Bijenička cesta 54, 10 000 Zagreb, Croatia; 4 Laboratory for Chemical and Biological Crystallography, Department of Physical Chemistry, Rudjer Bošković Institute, Bijenička cesta 54, 10 000 Zagreb, Croatia

**Keywords:** [3 + 2] cycloaddition, isoindoles, nitrile imines, pyrazoles, sydnones

## Abstract

New *trans*- and *cis*-*o*-stilbene-methylene-sydnones **3a**,**b** were synthesized by transforming the *trans*- and *cis*-*o*-aminomethylstilbene derivative, obtained by reduction of corresponding *o*-cyano derivatives, into glycine ester derivatives (43 and 31% yield) followed by hydrolysis (90 and 96% yield), nitrosation and ring closure with acetic acid anhydride (30 and 40% yield). The products were submitted to photochemical and thermal intramolecular [3 + 2] cycloadditions to afford diverse heteropolycyclic compounds. Photochemical reactions afforded *cis*-3-(4-methylphenyl)-3a,8-dihydro-3*H*-pyrazolo[5,1-*a*]isoindole (**11**, 12.5% yield) and *trans*-3-(4-methylphenyl)-3a,8-dihydro-3*H*-pyrazolo[5,1-*a*]isoindole (**12**, 5% yield). Thermal reactions afforded 3-(4-methylphenyl)-3,3a,8,8a-tetrahydroindeno[2,1-*c*]pyrazole (**14**, 50% yield) and 11-(4-methylphenyl)-9,10-diazatricyclo[7.2.1.0^2,7^]dodeca-2,4,6,10-tetraene (**15**, 22% yield).

## Introduction

**Figure 1:**
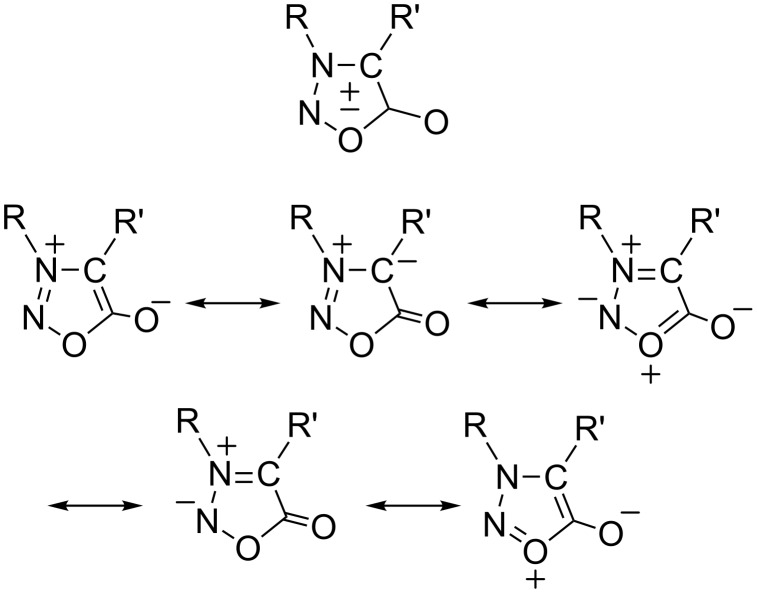
Resonance structures of the sydnone ring.

Sydnones belong to the group of five-membered heterocyclic compounds referred to as being "mesoionic" and have been widely studied since their discovery [1–5]. They can be represented as hybrids of a number of mesomeric ionic structures (Figure 1).

**Scheme 1:**
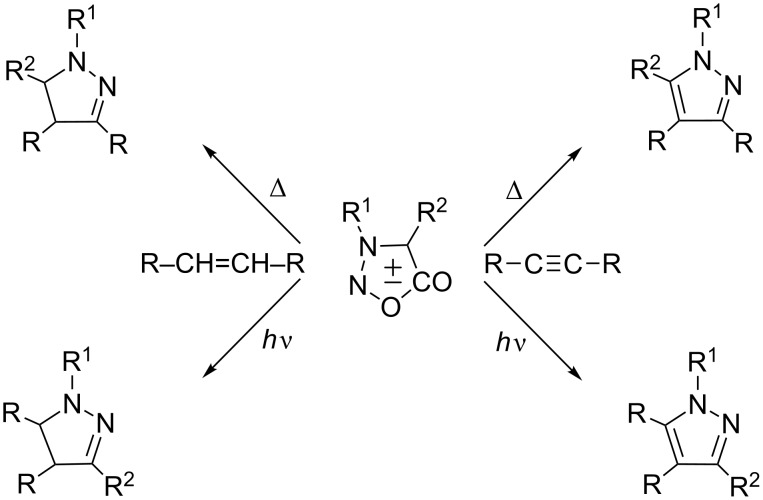
Thermal and photochemical intermolecular [3 + 2] cycloadditions.

One of the most characteristic reactions of sydnones is the intermolecular 1,3-dipolar cycloaddition. In the presence of acetylenic or ethylenic dipolarophiles, sydnones undergo cycloaddition reactions, which can be induced thermally [4,6–7] or photochemically [8–17], giving different pyrazole and/or pyrazoline derivatives, depending on the applied dipolarophile (Scheme 1). Namely, sydnones are masked 1,3-dipoles that by photolysis give nitrile imine intermediates, or in thermal reactions react as cyclic azomethine imines.

**Figure 2:**
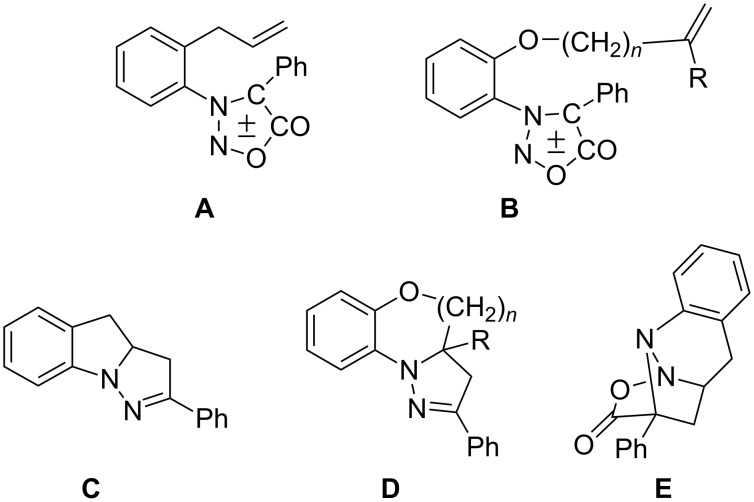
Illustration of intramolecular [3 + 2] cycloadditions.

Intramolecular 1,3-dipolar cycloadditions of sydnone derivatives have not been as thoroughly investigated, and so far only a few examples are known [18–20]. Photochemically induced intramolecular 1,3-dipolar cycloadditions have been studied on 3,4-disubstituted sydnone derivatives [18–19] (Figure 2, **A** and **B**), wherein indolopyrazole and pyrazolobenzoxazine structures are formed (Figure 2, **C** and **D**).

Heimgartner and coworkers also carried out the thermally induced reaction of 3-(*o*-allylphenyl)-4-phenylsydnone (**A**) and obtained the cycloadduct **E** (Figure 2) with the oxycarbonyl group remaining in the structure [18].

**Figure 3:**
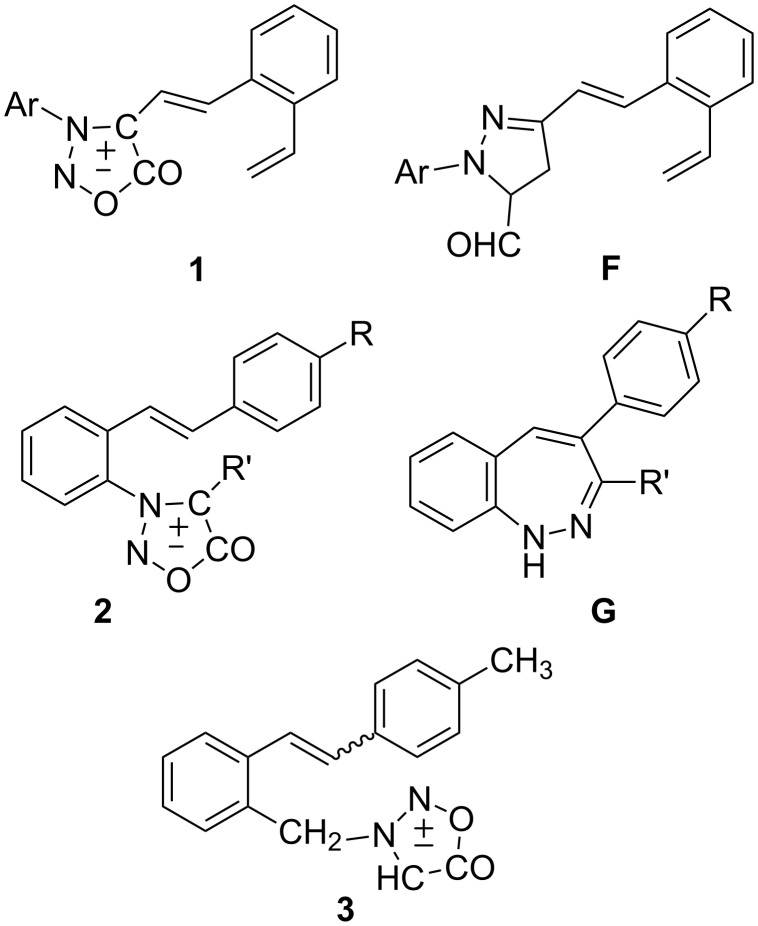
Styryl-sydnone **1** and stilbenyl sydnone **2** and their photoproducts **F** and **G**, respectively; target molecules **3** in this work.

We have been studying photochemical reactions of conjugated heterostilbene derivatives in which the sydnone moiety is part of a heterostilbene [17] (**1**, Figure 3) or is directly attached at the *ortho* position to the stilbene **2** [21–23]. Upon photolysis of compound **1**, where the sydnone moiety is part of a heterostilbene system, *cis*–*trans* isomerization was the main process, and no intramolecular cycloadducts were found owing to the unfavourable conformation of the formed intermediate in the *trans* configuration. The existence of the nitrile imine intermediate as a result of competitive photolysis of the sydnone moiety was confirmed on irradiation of **1** in the presence of acrolein and isolation of the pyrazoline derivative (**F**, Figure 3) [17]. In the case of stilbenylsydnones **2**, where the sydnone moiety is directly connected to the *ortho* position of the stilbene, the cyclization of the formed nitrile imine intermediate leading to benzodiazepine ring closure (**G**, Figure 3) was the main intramolecular process [22]. In a continuation of our interest in the synthesis of heteropolycyclic compounds we extended our research to new stilbene-sydnone derivatives **3** (Figure 3). In such a system, where two chromophores, stilbene and sydnone, are divided by a methylene bridge, an intramolecular 1,3-dipolar cycloaddition and the formation of diverse polycyclic compounds could be expected. Herein we describe, for the first time, the synthesis of *cis*- and *trans*-3-(stilbenylmethyl)sydnones and their photochemical and thermal intramolecular transformations to heteropolycyclic structures.

## Results and Discussion

**Scheme 2:**
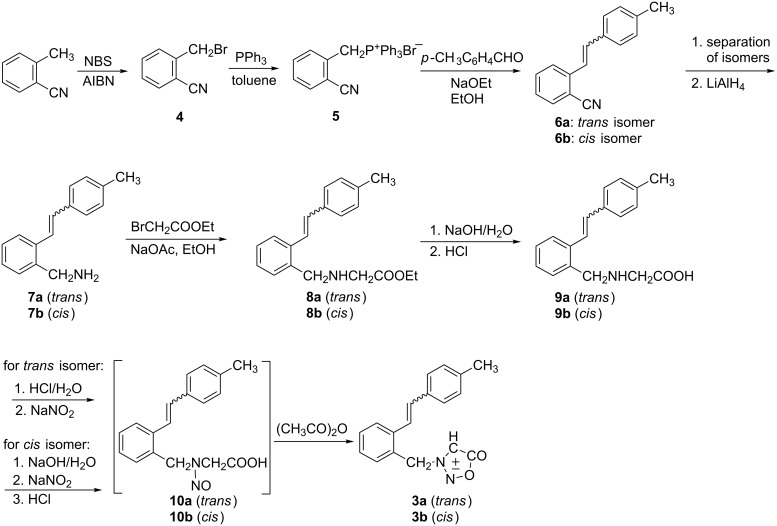
Synthesis of the target molecules **3a** and **3b**.

In the investigation of 3-{2-[2-(4-tolyl)ethenyl]phenyl}methylsydnones, **3a** (*trans*) and **3b** (*cis*), were prepared by a sequence of reactions (Scheme 2) starting from *o*-cyanotoluene (see Supporting Information File 1 for full experimental data). Bromination of *o*-cyanotoluene afforded 2-(bromomethyl)benzonitrile (**4**) [24], which was transformed to triphenylphosphonium salt **5** [25] followed by Wittig reaction with 4-methylbenzaldehyde to 2-(4-methylstyryl)benzonitrile (**6a**,**b**) [26]. The product was obtained as a mixture of **6a** (*trans* isomer, 40%) and **6b** (*cis* isomer, 60%).

The isomers were separated by column chromatography and further treated separately to achieve the final products in *cis* and *trans* configurations. Reduction of **6a** (*trans*) or **6b** (*cis*) with LiAlH_4_ in anhydrous ether afforded amino derivative **7a** (*trans*, 94%) or **7b** (*cis*, 93%). In the ^1^H NMR spectra new signals from the methylene protons appeared at 3.88 ppm (**7a**) and 3.62 ppm (**7b**) confirming the reduction. By further nucleophilic substitution, from **7a** (*trans*) or **7b** (*cis*) and ethyl bromoacetate, the ester **8a** (*trans*) or **8b** (*cis*) was prepared. On purification by column chromatography, the byproducts, obtained by disubstitution reaction of the amino compound, were separated, and the pure **8a** (43%) or **8b** (31%) was isolated. The obtained esters showed the presence of the carbonyl group at ~1740 cm^−1^ in the IR spectra and the carbonyl carbon at ~172 ppm in the ^13^C NMR spectra. The esters **8a** or **8b** were hydrolysed to the amino acid **9a** (*trans*, 90%) or **9b** (*cis*, 96%). The obtained amino acids were transformed to *N*-nitroso glycine **10a** or **10b** and, without isolation or further purification, were submitted to dehydration with acetic acid anhydride to give sydnone **3a** (*trans*) or **3b** (*cis*). After column chromatography the pure **3a** (30%) or **3b** (42%) was isolated. The best indication that the sydnone structures were formed was given by the singlet at ~6 ppm in the ^1^H NMR spectrum, characteristic for the proton H-4 in the sydnone ring, as well as those in the ^13^C NMR spectrum, namely the CH and CO sydnone carbons at ~94 and ~169 ppm, respectively.

**Scheme 3:**
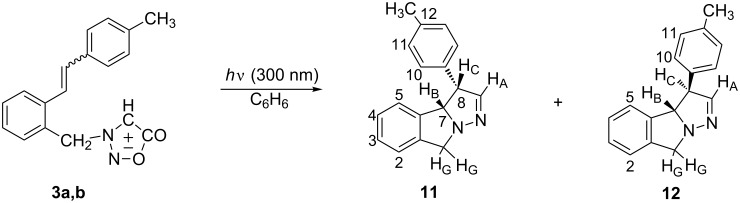
Photolysis of *cis*- or *trans*-**3**.

The irradiation experiments with the *trans* isomer (**3a**) or *cis* isomer (**3b**) were performed in ~10^−3^ M benzene solution in a Rayonet reactor at 300 nm under anaerobic conditions (purged with argon). The absorption maximum of *trans* isomer (**3a**) is at 300 nm (ε 37453) and of *cis* isomer (**3b**) at 291 nm (ε 16228), thus upon irradiation both isomers were excited. The irradiation of the isomers was performed until full conversion. The irradiation of either the *trans* or *cis* isomer, or of the mixture of isomers, resulted in the formation of two products in the same mutual ratio, along with large amounts of unidentified high-molecular-weight products. Separation by column chromatography in combination with thin layer chromatography gave dihydropyrazolo-isoindoles, **11** (12.5%) and **12** (5%) (Scheme 3).

The structure of the photoproducts was determined by spectroscopic methods. The molecular ions of **11** and **12**, *m*/*z* 248 in the mass spectra, indicate that the structures have lost CO_2_ relative to the starting compound. In the ^1^H and ^13^C NMR spectra, the signals are found in an area which is characteristic for saturated cyclic compounds. The major product is assigned to compound **11** based on the following data: In ^1^H NMR spectrum the signals at 4.98 ppm and 4.43 ppm are doublets with coupling constants of 15.6 Hz and are assigned to geminal protons G-1 and G-2. The other two signals at 4.92 and 4.24 ppm are also doublets but with coupling constants of 10.8 Hz. Based on the interaction of the proton at 5.88 with the proton at 4.92 ppm in the NOESY spectrum, the doublet at 5.88 is assigned to proton H-5, the doublet at 4.92 ppm to proton B and the doublet at 4.24 to proton C. Interaction of proton H-5 with proton B is also visible in HMBC spectrum. The signal at 6.53 ppm was assigned to proton A based on COSY interaction with proton C. The rather large high-field shift of the aromatic proton H-5 can be explained by an anisotropic effect of the tolyl group and thus confirms the *cis* orientation of protons B and C.

The structure of the minor photoproduct **12**, different from the structure **11** only in *trans* orientation of the protons B and C, was also evident from NMR spectra by using 2D NMR techniques. The doublets at 4.90 ppm and 4.37 ppm with a coupling constant of 15.6 Hz are assigned to geminal protons G. The singlet at 6.57 ppm is assigned to proton A. The chemical shift of proton A is the same as in structure **11**. The other two signals at 3.97 ppm and 4.70 ppm appear as singlets, and they are assigned to protons C and B, respectively, based on weak interactions in the COSY and NOESY spectra. Nevertheless, the proton H-5 of the fused benzene ring in structure **12** is in the multiplet together with other aromatic protons, which is in accordance with the proposed structure.

**Scheme 4:**
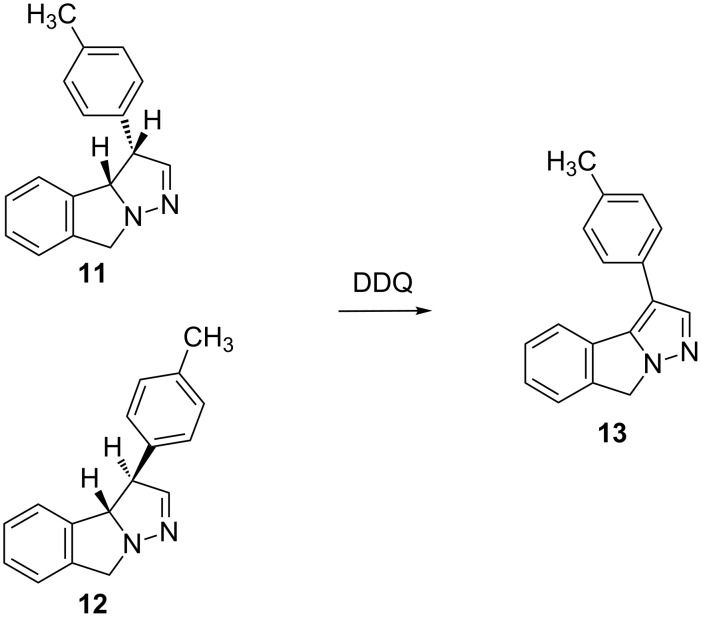
Aromatization with DDQ.

The structure of the photoproducts was confirmed by an additional experiment on the crude reaction mixture with DDQ (Scheme 4) in which, as expected for the predicted structures, the aromatization reaction took place forming the pyrazolo-isoindole **13**. Compound **13** arose also on silica gel during the purification of either **11** or **12**.

**Scheme 5:**
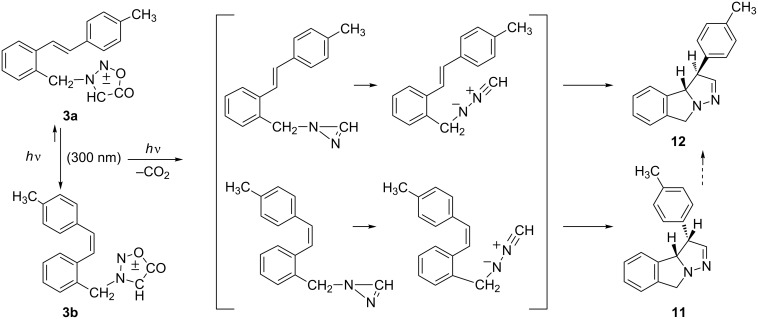
Possible mechanism for the formation of the photoproducts.

The irradiation of **3a** or **3b** until full conversion, as previously mentioned, produced a mixture of **11** and **12** along with decomposition and high-molecular-weight products. On shorter irradiation time (10 min, in benzene or acetonitrile) **3a** (*trans* isomer) afforded, according to ^1^H NMR, the photomixture of predominantly **3b** (*cis* isomer) with only traces of starting **3a** and tricyclic photoproducts **11** and **12**. Under the same irradiation conditions, **3b** (*cis* isomer) as starting compound gave a photomixture of *cis* isomer and the newly formed product **11** in 1:1 ratio with only traces of **3a** (*trans* isomer), along with some unidentified side products. The experimental results show that the *trans*-(**3a**) and *cis*-(**3b**) isomerize and react with different efficiency, and that the isomerization, as in the case of stilbene itself [27], is shifted toward the *cis* isomer. It follows that the reaction is stereospecific and that photoproduct **11** is formed from the *cis* configuration of the stilbene moiety and the photoproduct **12** from the *trans* configuration, although the formation of **12** via epimerization of **11** could not be eliminated. It is also evident that there are several competitive processes, which are summarized in Scheme 5.

On irradiation of **3a** (*trans*) or **3b** (*cis*) parallel competitive processes are in operation, namely, *trans*–*cis* and *cis*–*trans* isomerization of the stilbene moiety, and photolysis of the sydnone ring resulting in the formation of the nitrile imine intermediate. The nitrile imine species is, in intramolecular dipolar [3 + 2] cycloaddition, trapped by the *cis*- or *trans*-double bond of the stilbene, giving cycloadducts **11** or **12**, respectively.

We also performed the thermal intramolecular reactions with the starting compounds **3a** and **3b**. Theoretically the intramolecular 1,3-dipolar cycloaddition of the sydnone moiety, acting as a masked azomethine dipole, and the double bond of the stilbene moiety could proceed in different ways. The orientation of the sydnone ring toward the π bond of the stilbene in combination with the double bond configuration can give various formal [3 + 2] intramolecular cycloadducts.

**Scheme 6:**
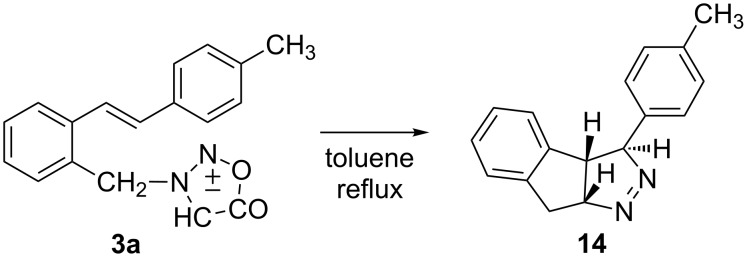
Thermal reaction of *trans*-**3**.

**Figure 4:**
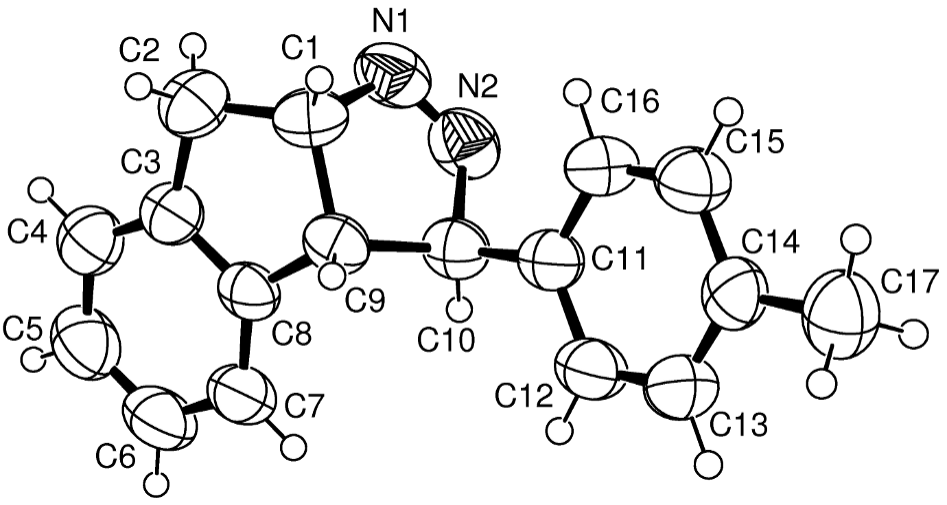
*ORTEP* of compound **14**.

On heating of **3a** (*trans*) in toluene until full conversion (4 h) one product in 50% yield was isolated from the reaction mixture after column chromatography. From the molecular ion (*m*/*z* 248) of the product and its ^13^C NMR spectrum it was obvious that in the cycloaddition CO_2_ elimination took place. Fragmentation of the product and the presence of an ion at *m*/*z* 220 suggests a structure in which the expulsion of nitrogen is possible. The structure **14** (Scheme 6) was determined by additional NMR techniques, NOE and HMBC interactions, and by single crystal X-ray structure analysis (Figure 4) of the crystal formed in an NMR tube by slow evaporation of the solvent.

**Scheme 7:**
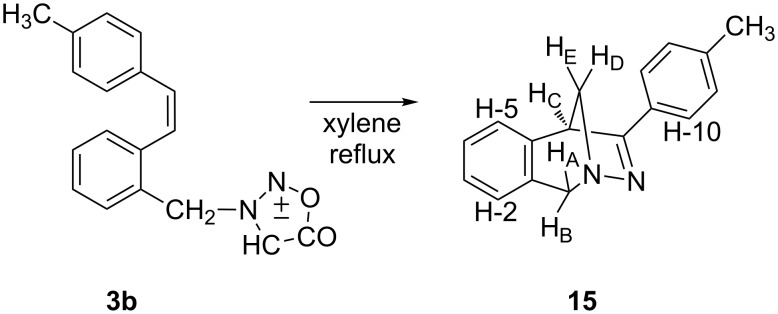
Thermal reaction of *cis*-**3**.

Also, when compound **3b** (*cis*) was refluxed in xylene (9 h) or toluene (19 h) only one cycloadduct **15** was isolated (Scheme 7), besides decomposition products, in 22% yield. The structure of **15** was determined by spectroscopic methods.

In the ^1^H NMR spectrum two pairs of geminal protons were found at 4.51 and 4.21 ppm (A, B) and at 3.71 and 3.35 ppm (E, D). The doublet at 3.95 ppm, coupled with one geminal proton (D), was assigned to proton C. In the ^13^C NMR spectrum, one of the five quaternary carbons is shifted to 176 ppm, which corresponds to an sp^2^-carbon in the vicinity of nitrogen. In the NOESY spectrum the interaction of protons A and B with an aromatic proton (H-2) at 7.00 ppm is seen, as well as the interaction of proton C with protons E and D. Since the NOE interaction is seen between protons A and E we concluded that protons A and E must lie on the same side of the six-membered ring. In addition, the interaction of proton C with tolyl (H-10) and H-5 protons was seen.

**Figure 5:**
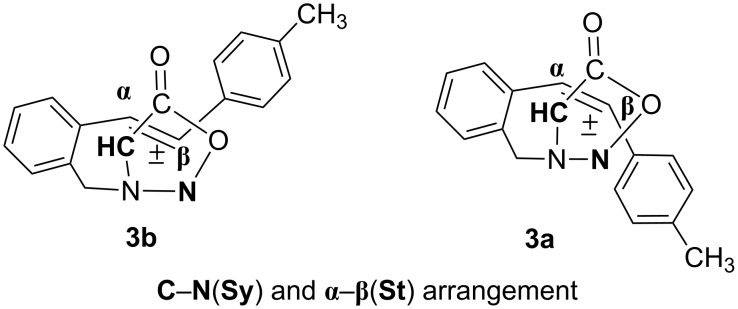
Proposed stereochemical pathway of sydnone ring (**CH**–**N**) and *trans*- and *cis*-stilbene (**α**–**β**).

**Figure 6:**
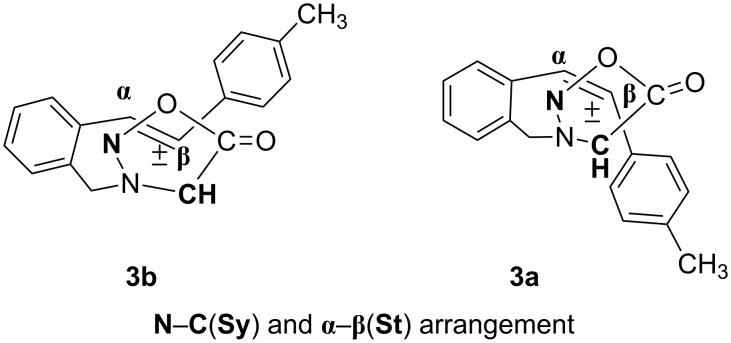
Proposed stereochemical pathway of sydnone ring (**N**–**CH**) and *trans*- and *cis*-stilbene (**α**–**β**).

In order to explain the diverse structures (**14** and **15**) and their formation mechanism, we analysed the possible ways of intramolecular [3 + 2] cycloaddition relating to the arrangement of the sydnone ring towards the *cis* and *trans* double bond (Figure 5 and Figure 6).

**Scheme 8:**
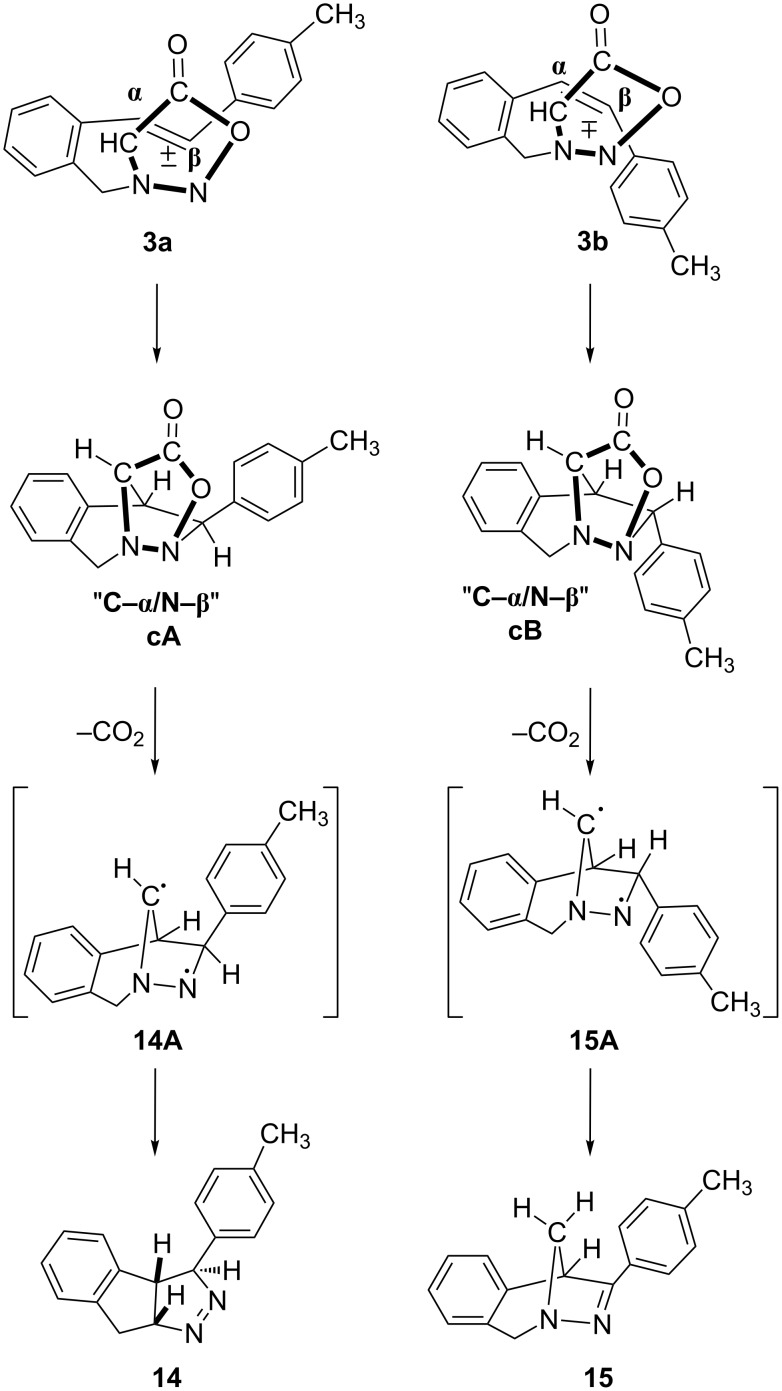
Possible formation of thermal products **14** (from *trans*-**3**) and **15** (from *cis*-**3**).

As presented in Figure 5, the sydnone ring could be oriented to the double bond in such a way that the bonds are formed at the C(Sy)–α(St) and N(Sy)–β(St) positions, or, as presented in Figure 6, at the N(Sy)–α(St) and C(Sy)–β(St) positions. The favoured arrangement of the sydnone ring toward the *cis* and *trans* double bond, leading to the products, is the pathway presented in Figure 5. The regiospecific and stereospecific formation of the products **14** and **15** could be explained by this approach of the sydnone ring (Scheme 8). The cycloadducts, **cA** from *trans* isomer and **cB** from *cis* isomer, lose CO_2_ under the reaction conditions to afford intermediates **14A** and **14 B**, respectively. Owing to the favourable conformation in the case of biradical **15A**, the 1,3-H abstraction and formation of the C–N double bond in product **15** is possible. In the biradical **14A** the intramolecular hydrogen abstraction is not favourable, but 1,2-alkyl shift takes place followed by formation of the N–N double bond in product **14**.

Monitoring the reaction by thin layer chromatography revealed that the [3 + 2] cycloaddition is much faster in the case of the *trans* isomer (**3a**). After the 4 h reflux of the toluene solution of the *trans* isomer, the ^1^H NMR spectrum of the crude reaction mixture showed complete conversion, while the *cis* isomer (**3b**) under the same conditions showed complete conversion only after 19 h. This evidence led us to believe that the formation of the "C–α/Ν–β" adduct **cA** proceeds via an energetically favoured transition state due to a possible secondary π–π interaction of the tolyl and carbonyl groups.

## Conclusion

In photochemical and thermal intramolecular reactions the investigated compounds **3a** and **3b**, in which the stilbene and sydnone ring are bridged by a methylene group, show the characteristic reaction for stilbene and sydnone moieties. The stilbene moiety photochemically isomerizes and the process of *trans*–*cis* isomerization is in competition with the photolysis of the sydnone ring. Photolysis of the sydnone moiety leads to a nitrile imine, followed by its intramolecular trapping by the *cis* or *trans* double bond of stilbene moiety, affording polycyclic compounds **11** and **12**, respectively. The same starting compounds also react thermally: The sydnone moiety in **3a** reacts as a masked azomethine dipole with *trans* configuration on the stilbene moiety by intramolecular [3 + 2] cycloaddition, giving polycyclic compound **14**, while the sydnone moiety in the *cis* isomer **3b** gives polycyclic compound **15**. Stilbene-methylene-sydnones are useful substrates for photochemical and thermal intramolecular [3 + 2] cycloaddition reactions to heteropolycyclic compounds.

## Supporting Information

File 1 Experimental details and characterization data for all compounds.

File 2 ^1^H NMR and APT spectra of **3a**, **3b**, **11**–**15**, NOESY spectra of **11**, **12**, **14** and **15** and X-ray data for **14**.
