# Supplementary material for: Photochemical and thermal intramolecular 1,3-dipolar cycloaddition reactions of new o-stilbene-methylene-3-sydnones and their synthesis
Source: Beilstein J Org Chem. 2011 Dec 13;7:1663–70. doi: 10.3762/bjoc.7.196 (PMC3252871; doi:10.3762/bjoc.7.196)
Supplement: File 1 — Experimental details and characterization data for all compounds. [file Beilstein_J_Org_Chem-07-1663-s001.pdf]

Supporting Information File 1

for

Photochemical and thermal intramolecular 1,3-dipolar cycloaddition reactions of new *o*-stilbene-methylene-3-sydnones and their synthesis

Kristina Butković[1,2], Željko Marinić[3], Krešimir Molčanov[4], Biserka Kojić-Prodić[4] and Marija Šindler-Kulyk*[1]

Address: [1]Department of Organic Chemistry, Faculty of Chemical Engineering and Technology, University of Zagreb, Marulićev trg 19, 10 000 Zagreb, Croatia, [2]Present address: Galapagos istraživački centar, Prilaz baruna Filipovića 29, 10 000 Zagreb, Croatia, [3]Center for NMR, Rudjer Bošković Institute, Bijenička cesta 54, 10 000 Zagreb, Croatia and [4]Laboratory for Chemical and Biological Crystallography, Department of Physical Chemistry, Rudjer Bošković Institute, Bijenička cesta 54, 10000 Zagreb, Croatia

Email: Marija Šindler-Kulyk - marija.sindler@fkit.hr

* Corresponding author

Experimental details and characterization data for all compounds

General

The ^{1}H and ^{13}C NMR spectra were recorded on a Bruker AV-600 Spectrometer at 300 and 600 MHz. All NMR spectra were measured in $CDCl_3$ or DMSO with tetramethylsilane as

reference. UV spectra were measured on a Varian Cary 50 UV/VIS Spectrophotometer. IR spectra were recorded on FTIR-ATR Vertex 70 Bruker or Perkin-Elmer M-297 spectrophotometer. Mass spectra were obtained on Extrel FT MS 2001 DD, Auto Spec Q (VG Analytical Manchester, GB), on Platform LCZ (Micromass, UK) and/or on a Varian Saturn 2200 equipped with Factor Four Capillary Column VF-5ms. Melting points were obtained by using an Original Kofler Mikroheitztisch apparatus (Reichert, Wien). Elemental analyses were carried out on Perkin-Elmer, Series II, CHNS Analyzer 2400 at Rudjer Bošković Institute. Silica gel (Merck 0.063e0.2 mm) was used for chromatographic purifications. Thin layer chromatography (TLC) was performed on Merck precoated silica gel 60 F254 plates. Solvents were purified by distillation. Single-crystal X-ray measurements were performed on an Enraf-Nonius CAD-4 diffractometer; details are given in Supporting Information File 2.

All benzene evaporations and work-up procedures with benzene were performed in the hood under good ventilation.

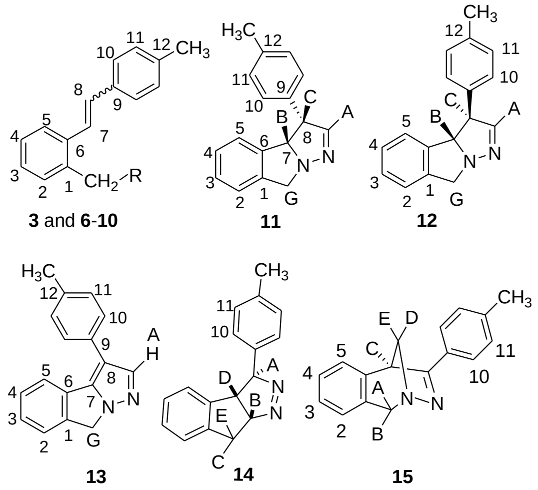

1. Synthesis of *trans*- and *cis*-3-(*o*-stilbenylmethyl)sydnones (3a, 3b)

1.1. Preparation of o-cyanobenzylbromide (**4**) [1]. The mixture of *o*-cyanotoluene (11.7 g, 0.10 mol), *N*-bromosuccinmide (19.6 g, 0.11mol), and 2,2'-azobis(2-methylpropionitrile) (0.6 g) in CCl_4 (100 mL) was heated under reflux for 1 h. The solid was filtered off after cooling and washed with CCl_4 (40 mL). Solvent evaporation afforded oily matter (19.3 g, 98%) which was used in the next reaction without any further purification.

1.2. Preparation of o-cyanobenzyltriphenylphosphonium bromide (**5**) [2]. Bromide **4** (19.3 g, 0.098 mol) was dissolved in toluene (40 mL) and solution of triphenylphosphine (26.2 g, 0.1 mol) in toluene (50 mL) was added. The reaction mixture was stirred at rt overnight and

then for 2 h at 50 °C. Filtration, washing out with petroleum ether (5 mL) and drying in a desiccator afforded the colourless solid of triphenylphosphonium bromide **5** (31.3 g, mp 237–241 °C, 70.2%).

1.3. Preparation of 2-cyano-4'-methylstilbene [3] (**6a, 6b**)

A solution of sodium ethoxide (0.345 g, 15.0 mmol of Na dissolved in 25 mL of ethanol) was added dropwise to a stirred solution of 2-cyanobenzyltriphenylphosphonium bromide (**5**, 5.59 g, 12.0 mmol) in absolute ethanol (50 ml) under a stream of argon. After that, 4-methylbenzaldehyde (1.20 g, 10.0 mmol) was added in small portions. Stirring was continued overnight at rt. After solvent removal, the residue was worked up with water and benzene. The benzene extracts were dried and concentrated. The crude reaction mixture was purified by column chromatography on silica gel using dichloromethane as eluent, affording a mixture of isomers (2.14 g, 97%, **6a**/**6b** in the ratio 3/2, based on ^{1}H NMR).

Repeating column chromatography on silica gel with petroleumether/diethylether (3:7) as eluent afforded the separation of isomers.

6a (*trans*): Yield 0.855 g (40%); colourless solid; mp 58–60 °C; UV (EtOH) λ_{max}/nm (ε/dm^{3} mol^{-1} cm^{-1}): 212 (22 738), 226 (13 666), 240 (13 521), 320 (25 111); IR (KBr) ν_{max}/cm^{-1}: 3027, 2922, 2854 (CH), 2220 (CN); ^{1}H NMR (300 MHz, $CDCl_3$) δ/ppm: 7.81 (d, 1H, *J* = 7.8 Hz, H-2), 7.66 (d, 1H, *J* = 7.8 Hz, H-5), 7.59 (t, 1H, *J* = 7.8 Hz, H-4), 7.63 (d, 2H, *J* = 8.0 Hz, H-10/11), 7.43 (d, 1H, *J* = 16.2 Hz, H-7/8), 7.33 (t, 1H, *J* = 7.8 Hz, H-3), 7.26 (d, 1H, *J* = 16.2 Hz, H-7/8), 7.22 (d, 2H, *J* = 8.0 Hz, H-10/11), 2.40 (s, 3H, CH_3); ^{13}C NMR (150 MHz, $CDCl_3$) δ/ppm: 140.76 (s), 138.95 (s), 133.41 (s), 133.33 (d), 133.11 (d), 132.78 (d), 129.61 (d, C-10/11), 127.36 (d), 127.11 (d, C-10/11), 125.12 (d), 122.99 (d), 118.15 (s, CN), 111.05 (s, C-1), 21.42 (q, CH_3); MS *m/z*: 219 (M^{+}, 100%).

6b (*cis*): Yield 1.283 g (60 %); colourless solid; mp 63–65 °C; UV (EtOH) λ_{max}/nm (ε/dm^{3} mol^{-1} cm^{-1}): 213 (34 011), 281 (9 297), 299 (9 295); IR (KBr) ν_{max}/cm^{-1}: 3016, 2918 (CH), 2222 (CN); ^{1}H NMR (600 MHz, $CDCl_3$) δ/ppm: 7.65 (d, 1H, *J* = 7.8 Hz, H-2/5), 7.37–7.36 (d, 2H, H-2/5 and H-3/4), 7.30 (m, 1H, H-4), 7.04 (d, 2H, *J* = 8.4 Hz, H-10/11), 7.03 (d, 2H, *J* = 8.4 Hz, H-10/11), 6.82 (d, 1H, *J* = 12.0 Hz, H-7/8), 6.72 (d, 1H, *J* = 12.0 Hz, H-7/8), 2.31 (s, 3H, CH_3); ^{13}C NMR (150 MHz, $CDCl_3$) δ/ppm: 141.45 (s), 137.63 (s), 134.22 (d), 132.94

(s), 132.77 (d), 132.01 (d), 129.61 (d), 128.96 (d, C-10/11), 128.73 (d, C-10/11), 127.21 (d), 125.07 (d), 117.78 (s), 112.02 (s), 21.10 (q, CH_3); MS *m*/*z*: 219 (M^+, 100%).

1.4. Preparation of trans- and cis-N-{{2-[2-(4-methylphenyl)ethenyl]phenyl}methyl}amine (**7a, 7b**)

$LiAlH_4$ (633 mg) was suspended in anhydrous diethyl ether (60 mL) and to that suspension was added **6a** or **6b (**2.42 g, 11.1 mmol) in small portions. The reaction mixture was heated at reflux for 1 h. After the mixture was cooled an additional amount of $LiAlH_4$ (400 mg) was added and the reaction mixture was stirred overnight at rt. The reaction was quenched with water (3 mL), the organic layer was separated and the aqueous layer was washed with diethyl ether (5 x 10 mL). Organic fractions were collected, dried ($MgSO_4$), filtered and concentrated, giving amino-derivative **7a** (*trans*) or **7b** (*cis*).

7a (*trans*): Yield 2.32 g (93.9%); colourless solid; mp 74–77 °C; UV (EtOH) λ_{max}/nm (ε/dm^3 mol^{-1} cm^{-1}): 206 (19 792), 227 (12 763), 300 (22 518); IR (KBr) ν_{max}/cm^{-1}: 3350, 3294 (NH_2), 2921, 2853 (CH); ^{1}H NMR (300 MHz, DMSO) δ/ppm: 7.65 (d, 1H, *J* = 9 Hz, H-2/5); 7.51 (d, 2H, *J* = 8.1 Hz, H-10/11), 7.47 (d, 1H, *J* = 16.2 Hz, H-7/8), 7.39 (d, 1H, *J* = 9 Hz, H-2/5), 7.26–7.23 (m, 2H, H-3 and H-4), 7.20 (d, 2H, *J* = 8.1 Hz, H-10/11), 7.08(d, 1H, *J* = 16.2 Hz, H-7/8), 3.88 (s, 2H, CH_2), 3.15 (bs, 2H, NH_2), 2.32 (s, 3H, CH_3); ^{13}C NMR (75 MHz, DMSO) δ/ppm: 141.00(s), 137.03 (s), 135.38 (s), 134.62 (s), 129.80 (d), 129.30 (d, C-10/11), 128.21 (d), 127.34 (d), 126.70 (d), 126.58, (d, C-10/11), 125.15 (d), 124.68 (d), 43.33 (t, CH_2), 20.88 (q, CH_3); MS *m*/*z*: 223 (M^+, 20.8%); Analysis calcd (%) for $C_{16}H_{17}N$ (Mr = 223.31): C, 86.05; H, 7.67; N, 6.27; found: C, 86.33; H, 7.47; N, 6.20.

7b (*cis*): Yield 2.31 g (93.8%); yellow oil; UV (EtOH) λ_{max}/nm (ε/dm^3 mol^{-1} cm^{-1}): 207 (23 789), 267 (9 875); IR (KBr) ν_{max}/cm^{-1}: 3330, 3323 (NH_2), 3013, 2919, 2862 (CH); ^{1}H NMR (600 MHz, DMSO) δ/ppm: 7.39 (d, 2H, *J* = 7.8 Hz, H-2/5), 7.17 (t, 1H, *J* = 7.8 Hz, H-3/4), 7.02 (t, 1H, *J* = 7.8 Hz, H-3/4), 6.95 (d, 2H, *J* = 7.8 Hz, H-2/5), 6.88 (s, 4H, H-10/11), 6.64 (d, 1H, *J* = 12.0 Hz, H-7/8), 6.54 (d, 1H, *J* = 12.0 Hz, H-7/8), 3.62 (s, 2H, CH_2), 3.05 (bs, 2H, NH_2), 2.13 (s, 3H, CH_3); ^{13}C NMR (150 MHz, DMSO) δ/ppm: 141.39 (s), 136.52 (s), 136.02 (s), 133.67 (s), 130.41 (d), 128.74 (d, C-10/11), 128.57 (d, C-10/11), 128.40 (d), 128.10 (d), 127.58 (d), 127.26 (d), 126.27 (d), 43.34 (t, CH_2), 20.74 (q, CH_3); MS *m*/*z*: 223 (M^+, 20.8%); Analysis calcd (%) for $C_{16}H_{17}N$ (Mr = 223.31): C, 86.05; H, 7.67; N, 6.27; found: C, 85.92; H, 7.85; N, 6.23.

1.5. Preparation of ethyl ester of trans- and cis-N-{{2-[2-(4-methylphenyl)ethenyl]phenyl}-methyl}glycine (**8a, 8b**)

A solution of **7a** (*trans*) or **7b** (*cis*) (2.18 g, 9.8 mmol), anhydrous sodium acetate (1.21 g, 14.7 mmol) and ethyl ester of α-bromoacetic acid (1.43 g, 8.8 mmol) in absolute ethanol (30 mL), was heated at reflux for 90 min. After the mixture was cooled, cold water (100 mL) was added to the reaction mixture under stirring and cooling (ice-bath). The reaction mixture was neutralized with $NaHCO_3$ and then extracted with diethyl ether (3 x 35 mL). Organic fractions were collected, dried ($MgSO_4$), filtered and concentrated affording a yellow oil, which was purified by column chromatography on silica gel with dichloromethane/ethanol (10/0.1) as eluent giving pure esters, **8a** (*trans*) or **8b** (*cis*).

8a (*trans*): Yield 1.30 g (43%); yellow oil; UV (EtOH) λ_{max}/nm (ε/dm^3 mol^{-1} cm^{-1}): 206 (27 868), 228 (17 300), 302 (29 663); IR (neat) ν_{max}/cm^{-1}: 3339 (NH), 2980 (CH), 1736 (CO); ^{1}H NMR (600 MHz, $CDCl_3$) δ/ppm: 7.64 (d, 1H, *J* = 7.8 Hz, H-2/5), 7.50 (d, 1H, *J* = 16.2 Hz, H-7/8), 7.47 (d, 2H, *J* = 8.1 Hz, H-10/11), 7.33 (d, 1H, *J* = 7.8 Hz, H-2/5), 7.30 (t, 1H, *J* = 7.8 Hz, H-3/4), 7.24 (t, 1H, *J* = 7.8 Hz, H-3/4), 7.18 (d, 2H, *J* = 8.1Hz, H-10/11), 7.02 (d, 1H, *J* = 16.2 Hz, H-7/8), 4.20 (q, 2H, *J* = 7.2 Hz, CH_2-est), 3.93 (s, 2H, CH_2), 3.46 (s, 2H, CH_2), 2.37 (s, 3H, CH_3), 1.27 (t, 3H, *J* = 7.2 Hz, CH_3-est); ^{13}C NMR (150 MHz, $CDCl_3$) δ/ppm: 172.25 (s, CO), 137.46 (s), 136.84 (s), 136.42 (s), 134.69 (s), 130.75 (d), 129.62 (d), 129.24 (d, C-10/11), 127.61 (d), 127.33 (d), 126.54 (d, C-10/11), 125.67 (d), 124.61 (d), 60.64 (t, CH_2), 50.89 (t, CH_2), 50.06 (t, CH_2), 21.12 (q, CH_3), 14.11 (q, CH_3-est); ^{1}H NMR (600 MHz, DMSO) δ/ppm: 7.64 (d, 1H, *J* = 7.8 Hz, H-2/5), 7.52 (d, 1H, *J* = 16.2 Hz, H-7/8), 7.47 (d, 2H, *J* = 7.8 Hz, H-10/11), 7.24 (d, 1H, *J* = 7.8 Hz, H-2/5), 7.22 (t, 1H, *J* = 7.8 Hz, H-3/4), 7.16 (t, 1H, *J* = 7.8 Hz, H-3/4), 7.12 (d, 2H, *J* = 7.8 Hz, H-10/11), 7.05 (d, 1H, *J* = 16.2 Hz, H-7/8), 4.03 (q, 2H, *J* = 7.2 Hz, CH_2-est), 3.78 (s, 2H, CH_2), 3.31 (s, 2H, CH_2), 2.24 (s, 3H, CH_3), 1.12 (t, 3H, *J* = 7.2 Hz, CH_3-est); ^{13}C NMR (150 MHz, DMSO) δ/ppm: 172.07 (s, CO), 137.13 (s), 136.94 (s), 136.23 (s), 134.57 (s), 129.69 (d), 129.62 (d), 129.18 (d, C-10/11), 127.28 (d), 127.12 (d), 126.53 (d, C-10/11), 125.05 (d), 124.66 (d), 59.92 (t, CH_2), 50.09 (t, CH_2), 49.61 (t, CH_2), 20.78 (q, CH_3), 14.07 (q, CH_3-est); MS *m/z*: 309 (M^+, 65.4%); Analysis calcd (%) for $C_{20}H_{23}NO_2$ (Mr = 309.4): C, 77.64; H, 7.49; N, 4.53; found: C, 77.80; H, 7.60; N, 4.52.

8b (*cis*): Yield 0.927g (30.6%); yellow oil; UV (EtOH) λ_{max}/nm (ε/dm^3 mol^{-1} cm^{-1}): 206 (31 734), 267 (13 503); IR (neat) ν_{max}/cm^{-1}: 3334 (NH), 3055, 3012, 2981, 292, 2686 (CH), 1737 (CO); ^{1}H NMR (600 MHz, $CDCl_3$) δ/ppm: 7.39 (d, 1H, *J* = 7.6 Hz, H-2/5), 7.24 (t, 1H, *J* = 7.6 Hz, H-3/4), 7.17 (d, 1H, *J* = 7.6 Hz, H-2/5), 7.14 (t, 1H, *J* = 7.6 Hz, H-3/4), 6.97 (d, 2H, *J* = 8.4 Hz, H-10/11), 6.95 (d, 2H, *J* = 8.4 Hz, H-10/11), 6.71 (d, 1H, *J* = 12.0 Hz, H-7/8), 6.61 (d, 1H, *J* = 12.0 Hz, H-7/8), 4.16 (q, 2H, *J* = 7.2 Hz, CH_2-est), 3.79 (s, 2H, CH_2), 3.38 (s, 2H, CH_2), 2.26 (s, 3H, CH_3), 1.24 (t, 3H, *J* = 7.2 Hz, CH_3-est); ^{13}C NMR (150 MHz, $CDCl_3$) δ/ppm: 172.16 (s, CO), 137.19 (s), 137.10 (s), 136.86 (s), 133.70 (s), 131.06 (d), 129.27 (d), 128.91 (d), 128.74 (d, C-10/11), 128.71 (d, C-10/11), 127.57 (d), 127.23 (d), 127.10 (d), 60.55 (t, CH_2), 50.90 (t, CH_2), 50.19 (t, CH_2), 21.02 (q, CH_3), 14.08 (q, CH_3-est); ^{1}H NMR (600 MHz, DMSO) δ/ppm: 7.35 (d, 1H, *J* = 7.8 Hz, H-2/5), 7.17 (t, 1H, *J* = 7.8 Hz, H-3/4), 7.04 (t, 1H, *J* = 7.8 Hz, H-3/4), 6.97 (d, 1H, *J* = 7.8 Hz, H-2/5), 6.89 (d, 2H, *J* = 8.4 Hz, H-10/11), 6.88 (d, 2H, *J* = 8.4 Hz, H-10/11), 6.65 (d, 1H, *J* = 12.0 Hz, H-7/8), 6.54 (d, 1H, *J* = 12.0 Hz, H-7/8), 3.98 (q, 2H, *J* = 7.2 Hz, CH_2-est), 3.63 (s, 2H, CH_2), 3.22 (s, 2H, CH_2), 2.13 (s, 3H, CH_3), 1.09 (t, 3H, *J* = 7.2 Hz, CH_3-est); ^{13}C NMR (150 MHz, DMSO) δ/ppm: 171.98 (s, CO), 137.93 (s), 136.77 (s), 136.49 (s), 133.65 (s), 130.40 (d), 128.71 (d, C-10/11), 128.63 (d, 2C), 128.56 (d, C-10/11), 128.02 (d), 127.18 (d), 126.69 (d), 59.89 (t, CH_2), 49.86 (t, CH_2), 49.74 (t, CH_2), 20.72 (q, CH_3), 14.10 (q, CH_3-est); MS *m/z*: 309 (M^+, 65.4%); Analysis calcd (%) for $C_{20}H_{23}NO_2$ (Mr = 309.4): C, 77.64; H, 7.49; N, 4.53; found: C, 77.84; H, 7.65; N, 4.46.

1.6. Preparation of trans- and cis-N-{{2-[2-(4-methylphenyl)ethenyl]phenyl}methyl}glycine (**9a**, **9b**)

A suspension of ester **8a** (*trans*) or **8b** (*cis*) (1.22 g, 4.0 mmol) and NaOH (192 mg, 4.8 mmol) dissolved in water (30 mL) was heated for 4 h under reflux after which time the reaction mixture was homogeneous. After cooling, the reaction mixture was extracted with diethyl ether (2 x 10 mL). The aqueous layer was acidified to pH 4.5 with concentrated hydrochloric acid. A white solid precipitated from the solution and was collected by filtration.

9a (*trans*): Yield 1.01g (90%); colourless solid; mp 168 °C; UV (EtOH) λ_{max}/nm (ε/dm^3 mol^{-1} cm^{-1}): 205 (22 851), 229 (13 599), 302 (23 424); IR (KBr) ν_{max}/cm^{-1}: ~3300–2500 (assoc. COOH), 3367 (NH), 2983, 2917 (CH), 1749 (CO); ^{1}H NMR (600 MHz, DMSO) δ/ppm: 7.69

(d, 1H, J = 7.8 Hz, H-2/5), 7.59 (d, 1H, J = 16.2 Hz, H-7/8), 7.53 (d, 2H, J = 7.8 Hz, H-10/11), 7.33 (d, 1H, J = 7.8 Hz, H-2/5), 7.28 (t, 1H, J = 7.8 Hz, H-3/4), 7.20 (t, 1H, J = 7.8 Hz, H-3/4), 7.11 (d, 2H, J = 7.8 Hz, H-10/11), 7.07 (d, 1H, J = 16.2 Hz, H-7/8), 4.02 (s, 2H, CH_2), 3.19 (s, 2H, CH_2), 2.23 (s, 3H, CH_3); ^{13}C NMR (150 MHz, DMSO) δ/ppm: 169.52 (s, CO), 137.20 (s), 136.88 (s), 134.51 (s), 133.30 (s), 130.74 (d), 130.69 (d), 129.25 (d, C-10/11), 128.43 (d), 127.37 (d), 126.93 (d, C-10/11), 125.36 (d), 124.17 (d), 49.50 (t, CH_2), 48.69 (t, CH_2), 20.93 (q, CH_3); MS *m/z*: 281 (M^+, 7.8%); Analysis calcd for $C_{18}H_{19}NO_2$ (Mr = 281.35): C, 76.84; H, 6.81; N, 4.98; found: C, 76.62; H, 6.82; N, 4.93.

9b (*cis*): Yield 1.08 g (96%); colourless solid; mp 199–200 °C; UV (EtOH) λ_{max}/nm (ε/dm^3 mol^{-1} cm^{-1}): 205 (24 107), 269 (9 817); IR (KBr) ν_{max}/cm^{-1}: ~3300–2500 (asoc. COOH), 3379 (NH), 2989, 2916 (CH), 1746 (CO); ^{1}H NMR (600 MHz, DMSO) δ/ppm: 9.50 (bs, 1H, OH), 7.64 (d, 1H, J = 7.8 Hz, H-2/5), 7.38 (t, 1H, J = 7.8 Hz, H-3/4), 7.31 (t, 1H, J = 7.8 Hz, H-3/4), 7.16 (d, 1H, J = 7.8 Hz, H-2/5), 7.00 (d, 2H, J = 7.8 Hz, H-10/11), 6.95 (d, 2H, J = 7.8 Hz, H-10/11), 6.83 (d, 1H, J = 12.0 Hz, H-7/8), 6.72 (d, 1H, J = 12.0 Hz, H-7/8), 4.15 (s, 2H, CH_2), 3.89 (s, 2H, CH_2), 2.23 (s, 3H, CH_3); ^{13}C NMR (150 MHz, DMSO) δ/ppm: 167.86 (s, CO), 138.06 (s), 136.90 (s), 133.11 (s), 131.91 (d), 130.39 (d), 129.65 (s), 129.26 (d), 129.02 (d), 128.80 (d, C-10/11), 128.67 (d, C-10/11), 127.64 (d), 126.92 (d), 47.25 (t, CH_2), 46.77 (t, CH_2), 20.70 (q, CH_3); MS *m/z*: 281 (M^+, 7.8%); Analysis calcd for $C_{18}H_{19}NO_2$ (Mr = 281.35): C, 76.84; H, 6.81; N, 4.98; found: C, 77.08; H, 6.58; N, 4.72.

1.7. Preparation of trans-3-{{2-[2-(4-methylphenyl)ethenyl]phenyl}methyl}sydnone (**3a**)

A suspension of **9a** (*trans*) (806 mg, 2.9 mmol) in water (30 mL) and HCl (1.0 mL, 17%) was cooled to 0 °C and stirred at the same temperature for 2 h. Then a solution of $NaNO_2$ (396 mg, 5.7 mmol) in water (2 mL) was added dropwise, and the reaction mixture was stirred with cooling at 0–4 °C for 2 h. Filtration and drying in a desiccator afforded crude nitroso **10a** (*trans*), which was dissolved in acetic anhydride (4 mL) and solution was heated for 90 min at 110 °C. After cooling, the reaction mixture was poured into water (150 mL) cooled in an ice-bath, the pH was adjusted to pH 9 by Na_2CO_3 addition and the product was extracted with dichloromethane (3 x 15 mL). Organic fractions were collected, dried ($MgSO_4$) and concentrated giving a yellow oil, which was purified by column chromatography on silica gel with dichloromethane as eluent, affording **3a** (*trans*).

3a (*trans*): Yield 257 mg (30.3%); colourless solid; mp 124–125 °C; UV (EtOH) λ_{max}/nm (ε/dm^3 mol^{-1} cm^{-1}): 204 (30 831), 227 (18 285), 300 (37 453); IR (KBr) ν_{max}/cm^{-1}: 3117 (CH-syd), 1751 (CO-syd); ^{1}H NMR (600 MHz, $CDCl_3$) δ/ppm: 7.71 (d, 1H, *J* = 7.8 Hz, H-5), 7.49 (m, 1H, H-4), 7.38 (d, 2H, *J* = 8.2 Hz, H-10/11), 7.36–7.35 (m, H-2 and H-3), 7.19 (d, 2H, *J* = 8.2 Hz, H-10/11), 7.18 (d, 1H, *J* = 15.9 Hz, H-7/8), 7.03 (d, 1H, *J* = 15.9 Hz, H-7/8), 6.10 (s, 1H, H-syd), 5.50 (s, 2H, CH_2), 2.3 (s, 3H, CH_3); ^{13}C NMR (75 MHz, $CDCl_3$) δ/ppm: 169.14 (s, CO-sid), 138.73 (s), 138.09 (s), 133.97 (d), 133.58 (s), 130.81 (d), 130.70 (d), 129.62 (d, C-10/11), 128.25 (d), 127.20 (s), 127.13 (d), 126.78 (d, C-10/11), 121.83 (d), 94.76 (d, C-syd), 55.18 (t, CH_2), 21.32 (q, CH_3); MS *m*/*z*: 292 (M^+, 5%); Analysis calcd for $C_{18}H_{16}N_2O_2$ (Mr = 292.33): C, 73.95; H, 5.52; N, 9.58; found: C, 73.88; H, 5.44; N, 9.51.

*1.8. Preparation of cis-3-{{2-[2-(4-methylphenyl)ethenyl]phenyl}methyl}sydnone (***3b***).*

A suspension of **9b** (*cis*) (456 mg, 1.0 mmol) and NaOH (44 mg, 1.9 mmol) dissolved in water (20 mL) was stirred for 1 h. Then a solution of $NaNO_2$ (144 mg, 2.0 mmol) in water (2 mL) was added dropwise and the reaction mixture was stirred at rt for 30 min. After that, the reaction mixture was cooled to 0 °C and HCl (17%) was added dropwise until pH 4.5 was achieved. The reaction mixture was stirred at the same temperature for 2 h. The product was extracted with benzene (3 x 30 mL). Organic fractions were collected, dried ($MgSO_4$), and concentrated giving crude nitroso **10b** (*cis*), which was dissolved in acetic anhydride (1.5 mL) and the solution was placed in the dark for 10 d. The reaction mixture was poured into water (100 mL) cooled in an ice-bath, the pH was adjusted to pH 9 by Na_2CO_3 addition and the product was extracted with benzene (3 x 15 mL). The organic fractions were collected, dried ($MgSO_4$) and concentrated, giving a yellow oil, which was purified by column chromatography on silica gel with dichloromethane as eluent to afford **3b** (*cis*).

3b (*cis*)*:* Yield 129 mg (42%); yellow oil; UV (EtOH) λ_{max}/nm (ε/dm^3 mol^{-1} cm^{-1}): 203 (30 087), 291 (16 228); IR (KBr) ν_{max}/cm^{-1}: 3124 (CH-syd), 3022, 2920 (CH), 1749 (CO-syd); ^{1}H NMR (600 MHz, $CDCl_3$) δ/ppm: 7.42 (t, 1H, *J* = 7.8 Hz, H-3/4), 7.36 (t, 1H, *J* = 7.8 Hz, H-3/4), 7.35 (d, 1H, *J* = 7.8 Hz, H-2/5), 7.29 (d, 1H, *J* = 7.8 Hz, H-2/5), 6.98 (d, 2H, *J* = 7.8 Hz, H-10/11), 6.89 (d, 2H, *J* = 7.8 Hz, H-10/11), 6.74 (d, 1H, *J* = 12.0 Hz, H-7/8), 6.59 (d, 1H, *J* = 12.0 Hz, H-7/8), 5.90 (s, 1H, H-syd), 5.22 (s, 2H, CH_2), 2.28 (s, 3H, CH_3); ^{13}C NMR (150 MHz, $CDCl_3$) δ/ppm: 168.70 (s, CO-syd), 137.84 (s), 137.68 (s), 133.09 (d), 132.37 (s), 129.86 (d), 129.81 (d), 129.24 (d), 128.78 (d, C-10/11), 128.26 (d, C-10/11), 127.85 (d),

127.51 (s), 124.82 (d), 94.31 (d, CH-syd), 54.39 (t, CH_2), 20.69 (q, CH_3); MS *m*/*z*: 292 (M^+, 5%); Analysis calcd for $C_{18}H_{16}N_2O_2$ (Mr = 292.33): C, 73.95; H, 5.52; N, 9.58; found: C, 74.13; H, 5.28; N, 9.86.

2. Irradiation experiments

2.1. Irradiation of the sydnone **3a**, **3b**

A solution of **3a** (*trans),* or **3b** (*cis)* or a mixture of **3a** and **3b** (50 mg, 0.17 mmol) in benzene (85 mL) was purged with argon for 15 min and irradiated in a quartz tube in a Rayonet reactor with 16 lamps of 300 nm for 30 min. Column chromatography combined with thin layer chromatography on silica gel (Silica gel 60 silanized) with dichloromethane as eluent afforded **12** (5%) and then **11** (12.5%).

trans-3-(4-Methylphenyl)-3a,8-dihydro-3H-pyrazolo[5,1-a]isoindole (**12**). Yield 2.1 mg (5%); oily matter; ^{1}H NMR δ/ppm (600 MHz, C_6D_6): 7.20–6.59 (m, 8H), 6.57 (s, 1H, A); 4.90 (d, 1H, *J* = 15.6 Hz, G-1), 4.70 (s, 1H, B), 4.37 (d, 1H, *J* = 15.6 Hz, G-2), 3.97 (s, 1H, C), 2.19 (s, 3H, CH_3); ^{13}C NMR δ/ppm (150 MHz, C_6D_6): 146.70 (d, C-A), 143.30 (s), 140.00 (s), 138.07 (s), 137.31 (s), 130.28 (d, C-10/11), 130.28 (d), 129.67 (d, C-11/10), 127.63 (d), 123.80 (d); 123.38 (d), 77.05 (d, C-B), 60.45 (d, C-C), 59.68 (t, C-G), 21.37 (q, CH_3); MS *m*/*z*: 248 (M^+, 100%); HRMS *m/z* for $C_{17}H_{16}N_2$: 248.1308; found: 248.1307.

cis-3-(4-Methylphenyl)-3a,8-dihydro-3H-pyrazolo[5,1-a]isoindole (**11**). Yield 5.3 mg (12.5%); oily matter; ^{1}H NMR (600 MHz, C_6D_6) δ/ppm: 6.92 (t, 1H, *J* = 7.2 Hz, H-3), 6.88 (d, 1H, *J* = 7.2 Hz, H-2), 6.85 (d, 2H, *J* = 7.8 Hz, H-11), 6.69 (t, 1H, *J* = 7.8 Hz, H-4), 6.64 (d, 2H, *J* = 7.8 Hz, H-10), 6.53 (s, 1H, A), 5.88 (d, 1H, *J* = 7.8 Hz, H-5), 4.98 (d, 1H, *J* = 15.6 Hz, G-1), 4.92 (d, 1H, *J* = 10.8 Hz, B), 4.43 (d, 1H, *J* = 15.6 Hz, G-2), 4.24 (d, 1H, *J* = 10.8 Hz, C), 2.08 (s, 3H, CH_3); ^{13}C NMR (150 MHz, C_6D_6) δ/ppm: 145.36 (d, C-A), 140.19 (s, C-1/6), 139.93 (s, C-6/1), 137.31 (s, C-12), 133.57 (s, C-9), 130.48 (d, C-10), 139.38 (d, C-11), 128.69 (d, C-3), 126.47 (d, C-4), 126.12 (d, C-5), 123.24 (d, C-2), 74.09 (d, C-B), 60.26 (t, C-G), 56.42 (d, C-C), 21.42 (q, CH_3); MS *m*/*z*: 248 (M^+, 100%); HRMS *m*/*z* for $C_{17}H_{16}N_2$: 248.1308; found: 248.1306.

2.2. Reaction with DDQ.

Compound **3a** (55 mg, 0.19 mmol) was dissolved in benzene (85 mL), purged with argon for 15 min and irradiated for 30 min in a Rayonet reactor with 16 lamps of 300 nm. The volume of the crude reaction mixture was then reduced to 20 mL and DDQ (43 mg, 0.19 mmol) was added. The reaction mixture was refluxed for 2 h. After concentration and purification by column chromatography on silica gel with dichloromethane as eluent, colourless solid of **13** was isolated.

3-(4-Methylphenyl)-8H-pyrazolo[5,1-a]isoindole (**13**): Yield 6 mg (23%); colourless solid; mp 137–139 °C; UV (EtOH) λ_{max}/nm (ε/dm^3 mol^{-1} cm^{-1}): 208 (26 857), 236 (10 892), 288 (12 688); IR (KBr) ν_{max}/cm^{-1}: 2920, 2864 (CH); ^{1}H NMR δ/ppm (300 MHz, $CDCl_3$): 7.80 (d, 1H, *J* = 7.5 Hz), 7.74 (s, 1H, A), 7.52 (d, 2H, *J* = 8.0 Hz, H-10/11), 7.49 (d, 1H, *J* = 7.5 Hz), 7.38 (t, 1H, *J* = 7.5 Hz), 7.33 (t, 1H, *J* = 7.5 Hz), 7.28 (d, 2H, *J* = 8.0 Hz, H-10/11), 5.16 (s, 2H, G), 2.41 (s, 3H); ^{13}C NMR δ/ppm (75 MHz, $CDCl_3$,): 142.81 (s), 142.38 (d), 140.79 (s), 136.55 (s), 131.36 (s), 130.38 (s), 129.78 (d), 128.38 (d), 127.52 (d), 127.51 (d), 123.75 (d), 120.58 (d), 116.16 (s), 52.32 (t, C-G), 21.40 (q); MS *m/z*: 246 (M$^+$, 100%); Analysis calcd for $C_{17}H_{14}N_2$ (Mr = 246.31): C, 82.90; H, 5.73; N, 11.37; found: C, 82.99; H, 5.44; N, 11.57.

3. Thermal reactions of 3-(*o*-stilbenylmethyl)sydnones

3.1. Thermal reaction of **3a** (*trans*)

Sydnone **3a** (*trans*) (15 mg, 0.05 mmol) was dissolved in toluene (10 mL) and heated under reflux for 4 h. Column chromatography on silica gel (Silica gel 60 silanized) with petroleum ether/dichloromethane as eluent afforded compound **14**.

3-(4-Methylphenyl)-3,3a,8,8a-tetrahydroindeno[2,1-c]pyrazole (**14**): Yield 6.2 mg (50%); colourless solid; mp 89–91 °C; UV (EtOH) λ_{max}/nm (ε/dm^3 mol^{-1} cm^{-1}): 204 (25 812), 260 (2 086), 266 (2 484), 317 (1 099); IR (KBr) ν_{max}/cm^{-1}: 3024, 2922, 2852 (CH); ^{1}H NMR (600 MHz, $CDCl_3$,) δ/ppm: 7.23–7.17 (m, 6H), 7.04 (d, 2H, *J* = 7.8 Hz, H-10), 5.85 (d, 1H, *J* = 1.8 Hz, A), 5.43 (ddd, 1H, *J* = 1.8, 7.2, 9.2 Hz, B), 3.68 (d, 1H, *J* = 16.8 Hz, C), 3.57 (d, 1H, *J* = 7.2 Hz, D), 3.52 (dd, 1H, *J* = 16.8, 9.2 Hz, E), 2.32 (s, 3H); ^{13}C NMR (150 MHz, $CDCl_3$) δ/ppm: 143.45 (s), 139.90 (s), 137.87 (s), 134.87 (s), 129.77 (d, C-11), 127.94 (d), 127.52 (d), 126.88 (d, C-10), 125.25 (d), 124.61 (d), 99.54 (d, C-A), 92.85 (d, C-B), 50.29 (d,

C-D), 35.19 (t, C-C/E), 21.13 (q, CH_3); ^{1}H NMR (600 MHz, C_6D_6) δ/ppm: 7.03–7.02 (m, 2H), 6.99 (d, 2H, *J* = 8.0 Hz, H-11), 6.94 (d, 2H, *J* = 8.0 Hz, H-10), 6.93–6.92 (m, 2H), 5.81 (d, 1H, *J* = 1.8 Hz, A), 4.90 (ddd, 1H, *J* = 1.8, 7.0, 9.5 Hz, B), 3.55 (d, 1H, *J* = 17.2 Hz, C), 3.19 (d, 1H, *J* = 7.0 Hz, D), 3.03 (dd, 1H, *J* = 17.2, 9.5 Hz, E), 2.11 (s, CH_3); ^{13}C NMR (150 MHz, C_6D_6) δ/ppm: 143.80 (s), 140.02 (s), 137.26 (s), 135.72 (s), 129.62 (d), 128.09 (d), 127.73 (d), 127.09 (d), 125.20 (d), 124.64 (d), 99.68 (d, C-A), 92.90 (d, C-B), 50.33 (d, C-D), 35.01 (t, C-C/E), 20.81 (q, CH_3); MS *m*/*z*: 248.13 (M^+, 5%); Analysis calcd (%) for $C_{17}H_{16}N_2$ (Mr = 248.33): C, 82.22; H, 6.49; N, 11.28; found: C, 82.50; H, 6.31; N, 11.19.

3.2. Thermal reaction of **3b** (*cis*)

Sydnone **3b** (44 mg, 0.15 mmol) was dissolved in toluene (30 mL) and heated at reflux for 19 h (or in xylene for 9 h). Column chromatography on silica gel with dichloromethane/ethanol/diethyl ether (5/0.1/0.1) as eluent afforded compound **15**.

11-(4-Methylphenyl)-9,10-diazatricyclo[7.2.1.0^{2,7}]dodeca-2,4,6,10-tetraene (**15**). Yield 7.4 mg (22%); colourless solid; mp 131–133 °C; UV (EtOH) λ_{max}/nm (ε/dm^3 mol^{-1} cm^{-1}): 206 (26 039), 248 (10 181), 286 (5 221); IR (KBr) ν_{max}/cm^{-1}: 2924 (CH); ^{1}H NMR (600 MHz, $CDCl_3$) δ/ppm: 7.78 (d, 2H, *J* = 8.1 Hz, H-10), 7.21–7.17 (m, 4H, H-11, H-5, H-3), 7.11 (t, 1H, *J* = 7.4 Hz, H-4), 7.00 (d, 1H, *J* = 7.4 Hz, H-2), 4.51 (d, 1H, *J* = 17.5 Hz, A), 4.21 (d, 1H, *J* = 17.5 Hz, B), 3.95 (d, 1H, *J* = 3.8 Hz, C), 3.71 (dd, 1H, J_{DE} = 10.6 Hz, J_{DC} = 3.8 Hz, D), 3.35 (d, 1H, *J* = 10.6 Hz, E), 2.35 (s, 3H, CH_3); ^{13}C NMR (150 MHz, $CDCl_3$) δ/ppm: 176.03 (s), 141.63 (s), 136.98 (s), 131.76 (s), 129.64 (d, C-11), 128.60 (d), 128.18 (d, C-4), 127.98 (d, C-10), 127.72 (s), 126.19 (d, C-2), 125.95 (d), 58.04 (t, C-DE), 52.24 (t, C-AB), 47.10 (d, C-C), 21.69 (q, CH_3); MS *m*/*z*: 249 (M^++1, 100%); Analysis calcd (%) for $C_{17}H_{16}N_2$ (Mr = 248.33): C, 82.22; H, 6.49; N, 11.28; found: C, 82.10; H, 6.78; N, 11.12.

References

1. Gorsane, M; Defay, N; Martin, R. H. *Bull. Soc. Chim. Belg*. **1985**, *94*, 215-231.

2. Palmer, B. D.; Thompson, A. M.; Booth, R. J.; Dobrusin, E. M.; Kraker, A. J.; Lee, H. H.; Lunney, E. A.; Mitchell, L. H.; Ortwine, D. F.; Smaill, J. B.; Swan, L. M.; Denny, W. A. *J. Med. Chem*. **2006**, **49**, 4896-4911.

3. Husemoen, G.; Olsson, R.; Andersson, C.-M.; Harvey, S. C.; Hansen, H. C. *J. Comb. Chem*. **2003**, *5*, 606-609 (the isomers are not separated).
