# Supplementary material for: Photochemical and thermal intramolecular 1,3-dipolar cycloaddition reactions of new o-stilbene-methylene-3-sydnones and their synthesis
Source: Beilstein J Org Chem. 2011 Dec 13;7:1663–70. doi: 10.3762/bjoc.7.196 (PMC3252871; doi:10.3762/bjoc.7.196)
Supplement: File 2 — 1H NMR and APT spectra of 3a, 3b, 11–15, NOESY spectra of 11, 12, 14 and 15 and X-ray data for 14. [file Beilstein_J_Org_Chem-07-1663-s002.pdf]

Supporting Information File 2

for

Photochemical and thermal intramolecular 1,3-dipolar cycloaddition reactions of new *o*-stilbene-methylene-3-sydnones and their synthesis

Kristina Butković[1,2], Željko Marinić[3], Krešimir Molčanov[4], Biserka Kojić-Prodić[4] and Marija Šindler-Kulyk*[1]

core

Address: [1]Department of Organic Chemistry, Faculty of Chemical Engineering and Technology, University of Zagreb, Marulićev trg 19, 10 000 Zagreb, Croatia, [2]Present address: Galapagos istraživački centar, Prilaz baruna Filipovića 29, 10 000 Zagreb, Croatia, [3]Center for NMR, Rudjer Bošković Institute, Bijenička cesta 54, 10 000 Zagreb, Croatia and [4]Laboratory for Chemical and Biological Crystallography, Department of Physical Chemistry, Rudjer Bošković Institute, Bijenička cesta 54, 10000 Zagreb, Croatia

Email: Marija Šindler-Kulyk - marija.sindler@fkit.hr

* Corresponding author

^{1}H NMR and APT spectra of 3a, 3b, 11–15, NOESY spectra of 11, 12, 14 and 15 and X-ray data for 14.

Contents:

1. ^{1}H NMR and APT spectra of
   trans-3-{{2-[2-(4-methylphenyl)ethenyl]phenyl}methyl}sydnone (**3a**) S3

2. ^{1}H NMR and APT spectra of
   cis-3-{{2-[2-(4-methylphenyl)ethenyl]phenyl}methyl}sydnone (**3b**)............................... S4

3. ^{1}H NMR and APT spectra of
   cis-3-(4-methylphenyl)-3a,8-dihydro-3*H*-pyrazolo[5,1-a]isoindole (**11**) S5

4. ^{1}H NMR and APT spectra of
   trans-3-(4-methylphenyl)-3a,8-dihydro-3*H*-pyrazolo[5,1-a]isoindole (**12**)....................... S6

5. ^{1}H NMR and APT spectra of
   3-(4-methylphenyl)-8*H*-pyrazolo[5,1-a]isoindole (**13**) S7

6. ^{1}H NMR and APT spectra of
   3-(4-methylphenyl)-3,3a,8,8a-tetrahydroindeno[2,1-c]pyrazole (**14**) S8

7. ^{1}H NMR and APT spectra of
   11-(4-methylphenyl)-9,10-diazatricyclo[7.2.1.0^{2,7}]dodeca-2,4,6,10-tetraene (**15**) S9

8. NOESY spectrum of **11**.. S10

9. NOESY spectrum of **12**.. S10

10. NOESY spectrum of **14**.. S11

11. NOESY spectrum of **15**.. S11

12. X-Ray data... S12

13. References S13

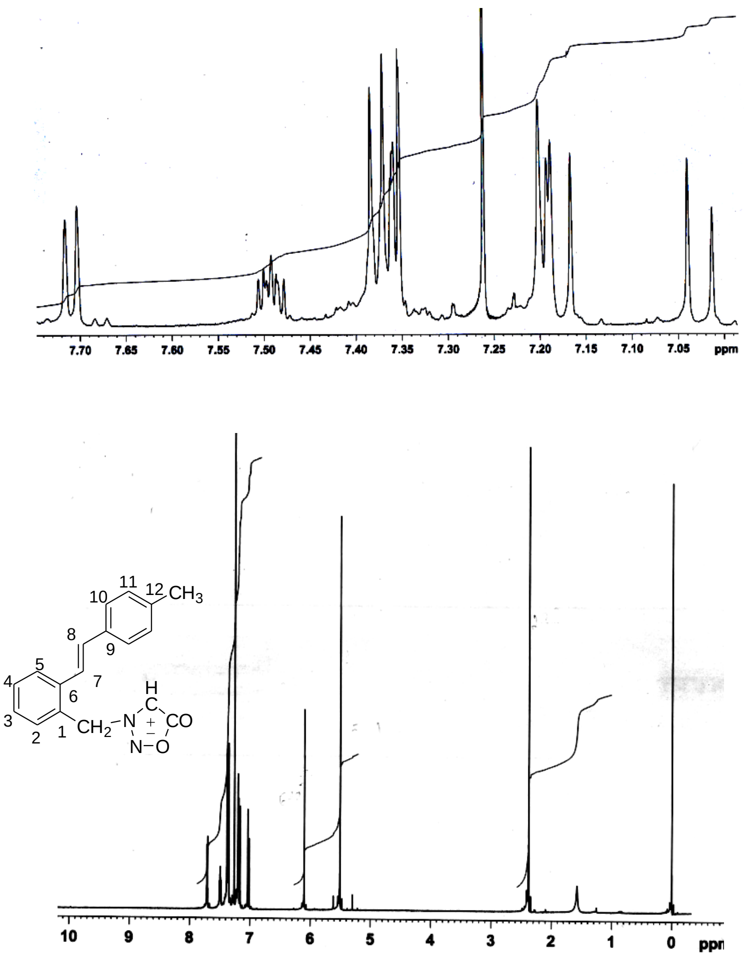

Figure 1: ^{1}H NMR spectrum (600 MHz, $CDCl_3$) of *trans*-3-{{2-[2-(4-methylphenyl)ethenyl]phenyl}methyl}sydnone (**3a**).

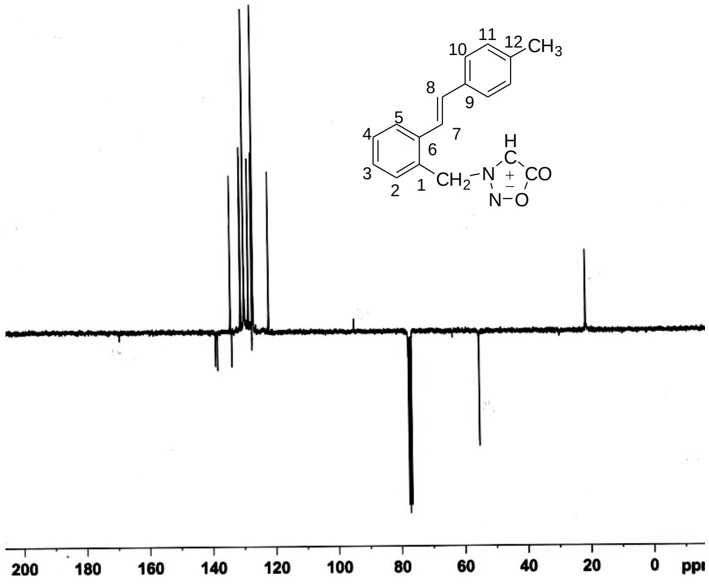

Figure 2: APT spectrum (150 MHz, $CDCl_3$) of *trans*-3-{{2-[2-(4-methylphenyl)ethenyl]phenyl}methyl}sydnone (**3a**).

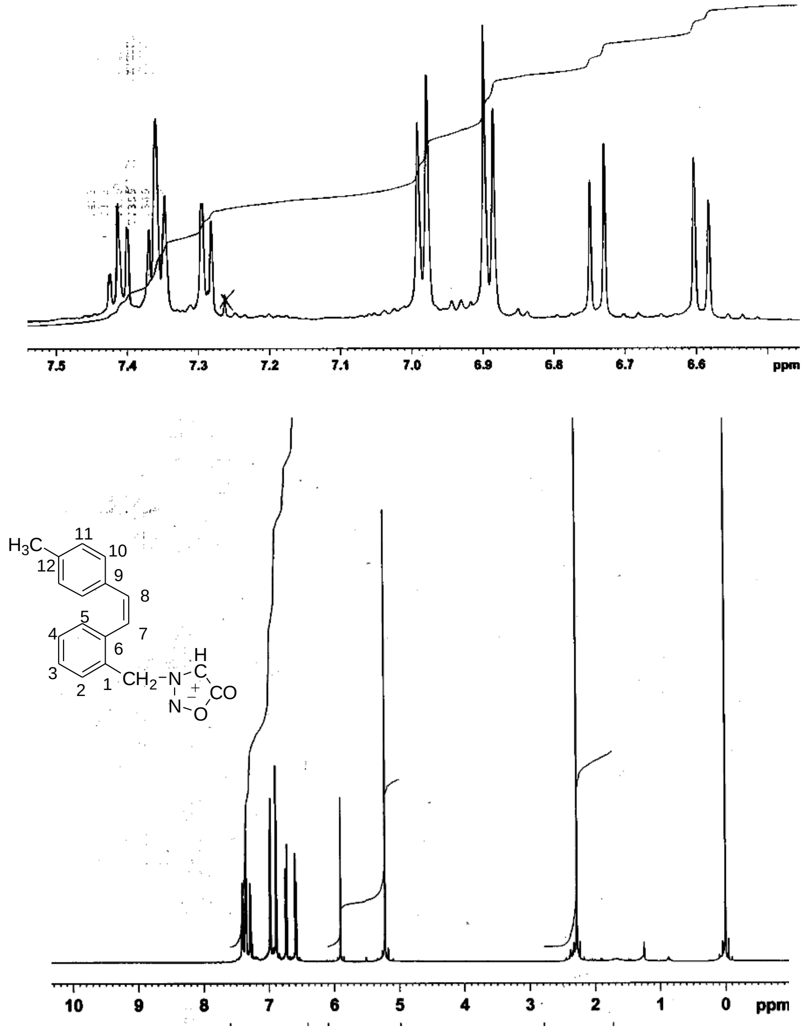

Figure 3: ^{1}H NMR spectrum (600 MHz, $CDCl_3$) of
cis-3-{{2-[2-(4-methylphenyl)ethenyl]phenyl}methyl}sydnone (**3b**).

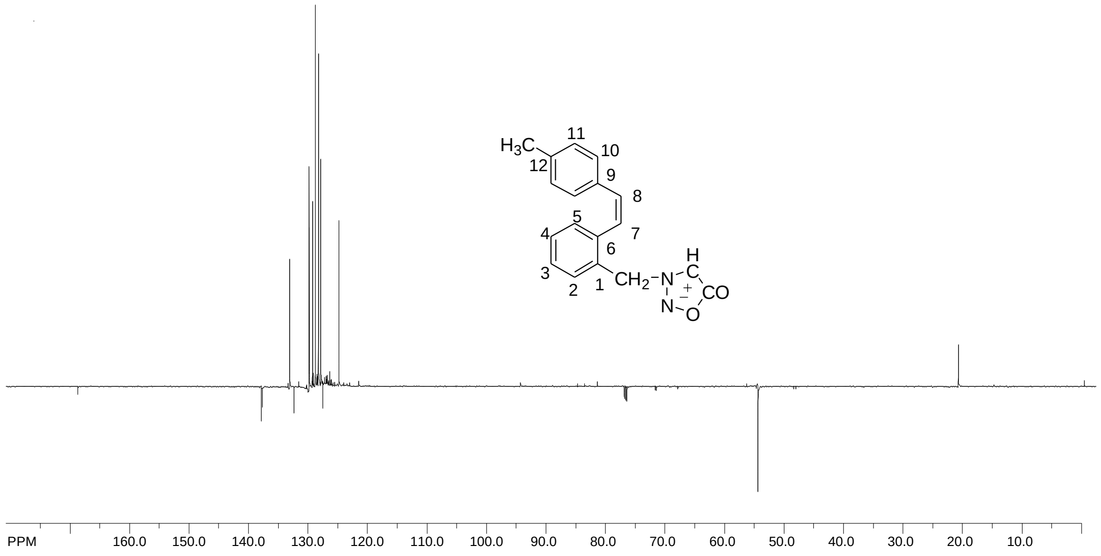

Figure 4: APT spectrum (150 MHz, $CDCl_3$) of
cis-3-{{2-[2-(4-methylphenyl)ethenyl]phenyl}methyl}sydnone (**3b**).

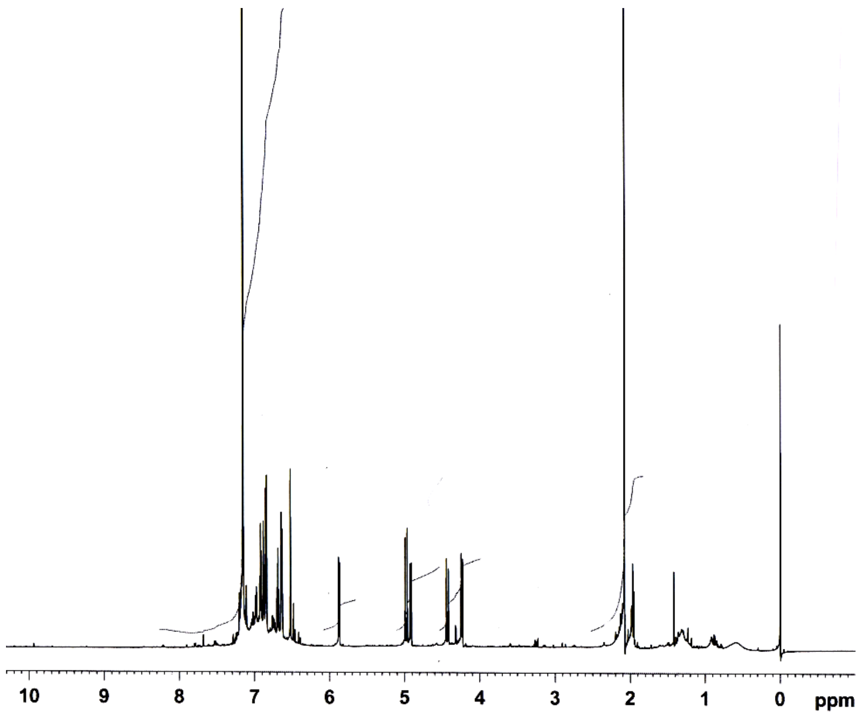

Figure 5: ^{1}H NMR spectrum (600 MHz, C_6D_6) of
cis-3-(4-methylphenyl)-3a,8-dihydro-3H-pyrazolo[5,1-a]isoindole (**11**).

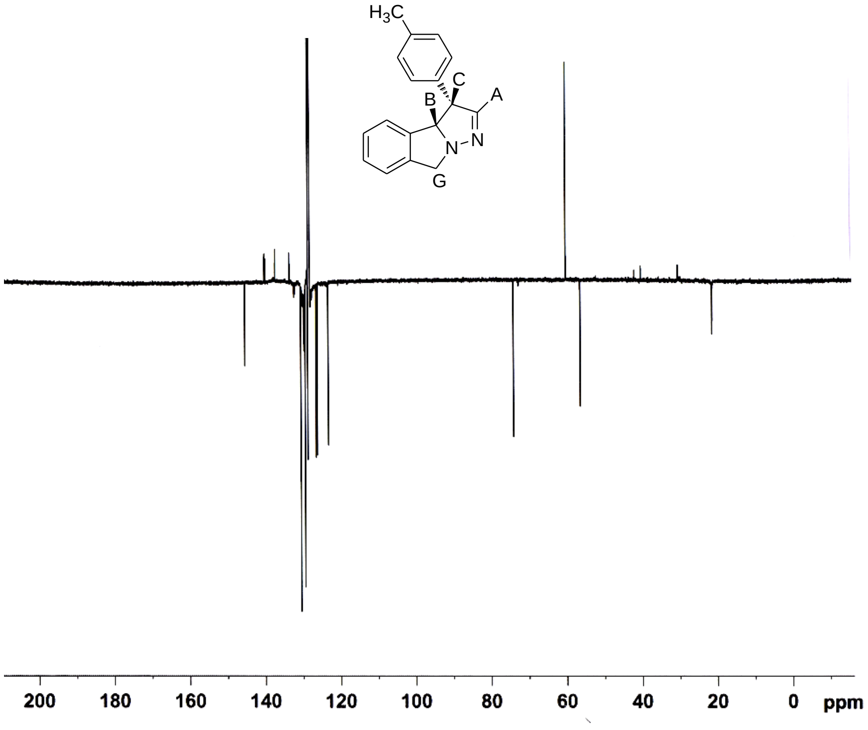

Figure 6: APT spectrum (150 MHz, C_6D_6) of
cis-3-(4-methylphenyl)-3a,8-dihydro-3H-pyrazolo[5,1-a]isoindole (**11**).

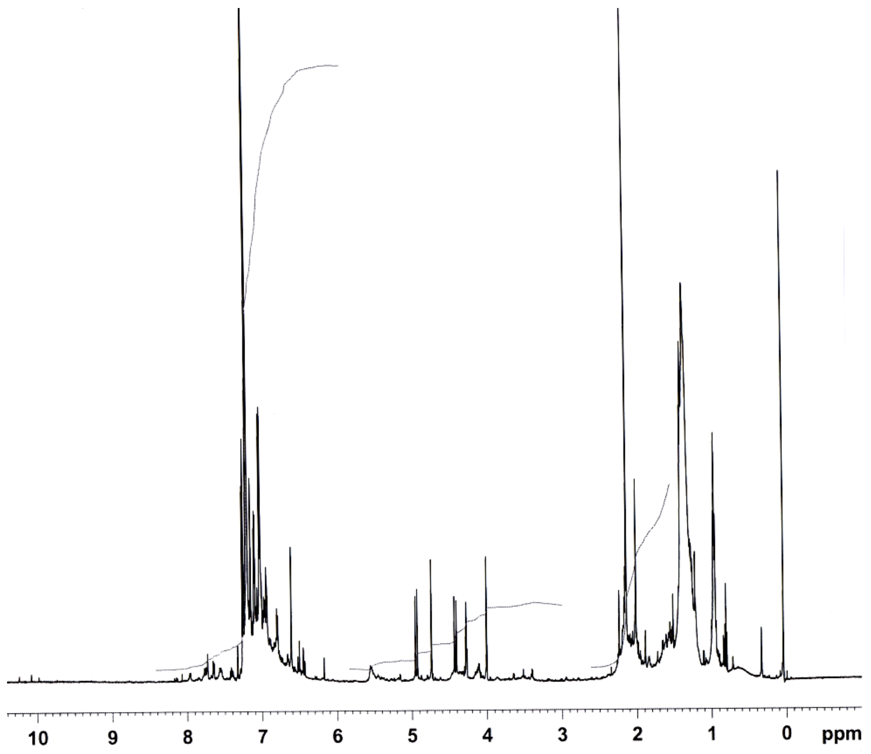

Figure 7: ^{1}H NMR spectrum (600 MHz, C_6D_6) of
trans-3-(4-methylphenyl)-3a,8-dihydro-3*H*-pyrazolo[5,1-a]isoindole (**12**).

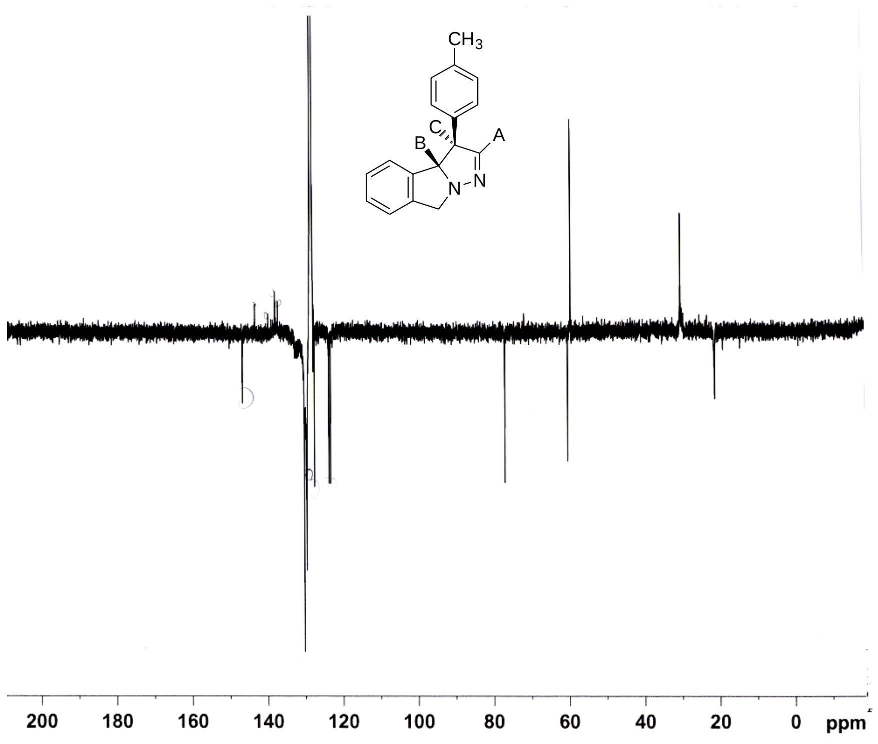

Figure 8: APT spectrum (150 MHz, C_6D_6) of
trans-3-(4-methylphenyl)-3a,8-dihydro-3*H*-pyrazolo[5,1-a]isoindole (**12**).

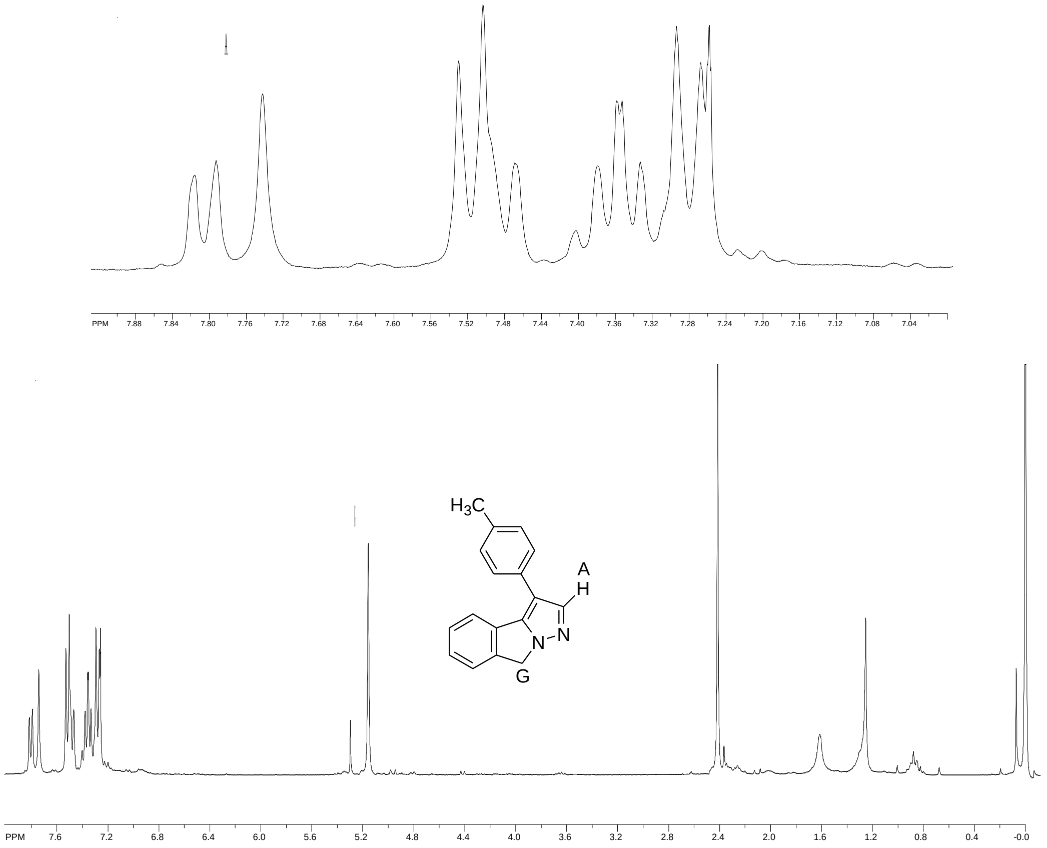

Figure 9: ^{1}H NMR spectrum (300 MHz, $CDCl_3$) of
3-(4-methylphenyl)-8*H*-pyrazolo[5,1-a]isoindole (**13**).

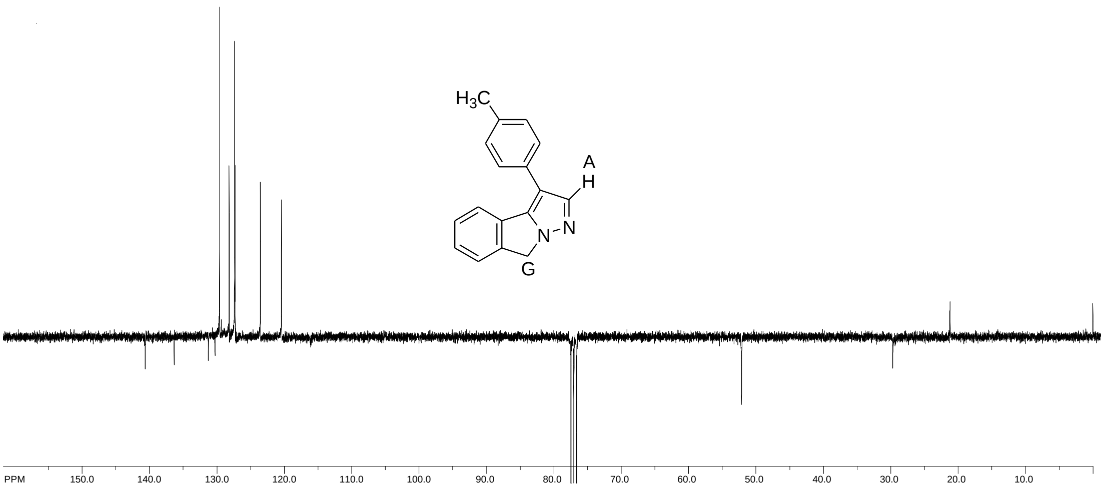

Figure 10: APT spectrum (75 MHz, $CDCl_3$) of
3-(4-methylphenyl)-8*H*-pyrazolo[5,1-a]isoindole (**13**).

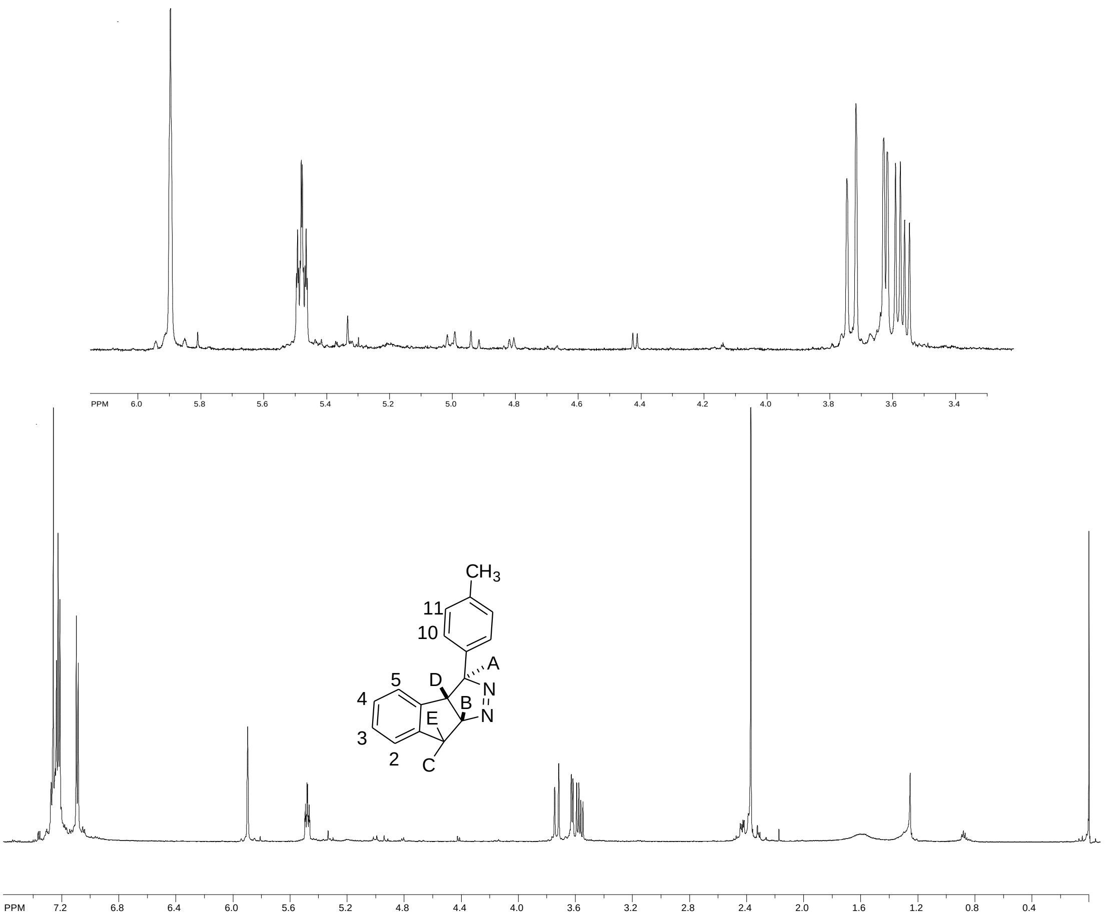

Figure 11: ^{1}H NMR spectrum (600 MHz, $CDCl_3$) of 3-(4-methylphenyl)-3,3a,8,8a-tetrahydroindeno[2,1-c]pyrazole (**14**)

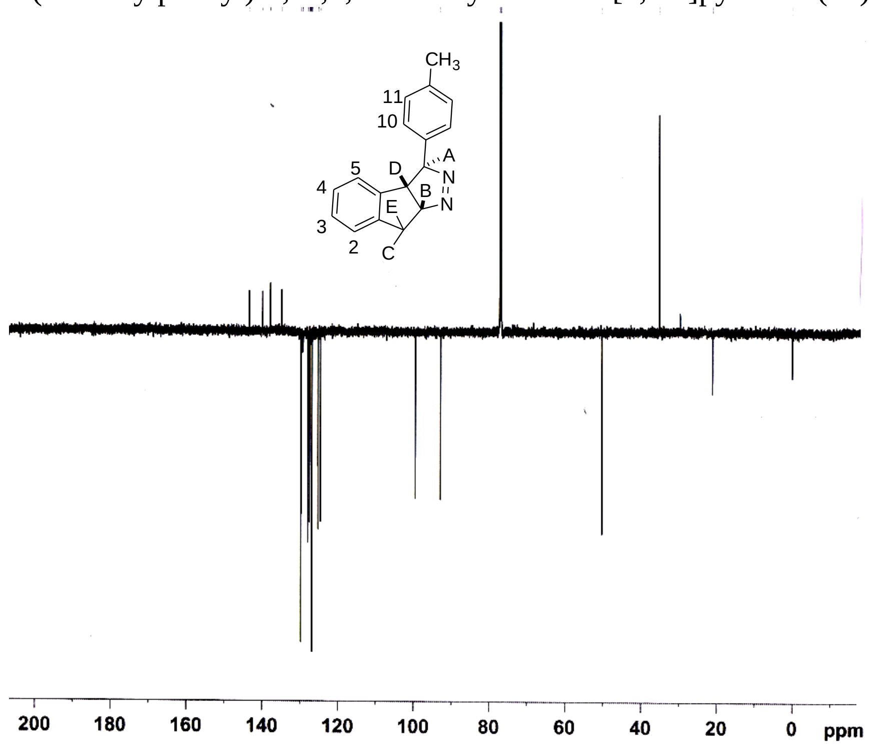

Figure 12: APT spectrum (150 MHz, $CDCl_3$) of 3-(4-methylphenyl)-3,3a,8,8a-tetrahydroindeno[2,1-c]pyrazole (**14**)

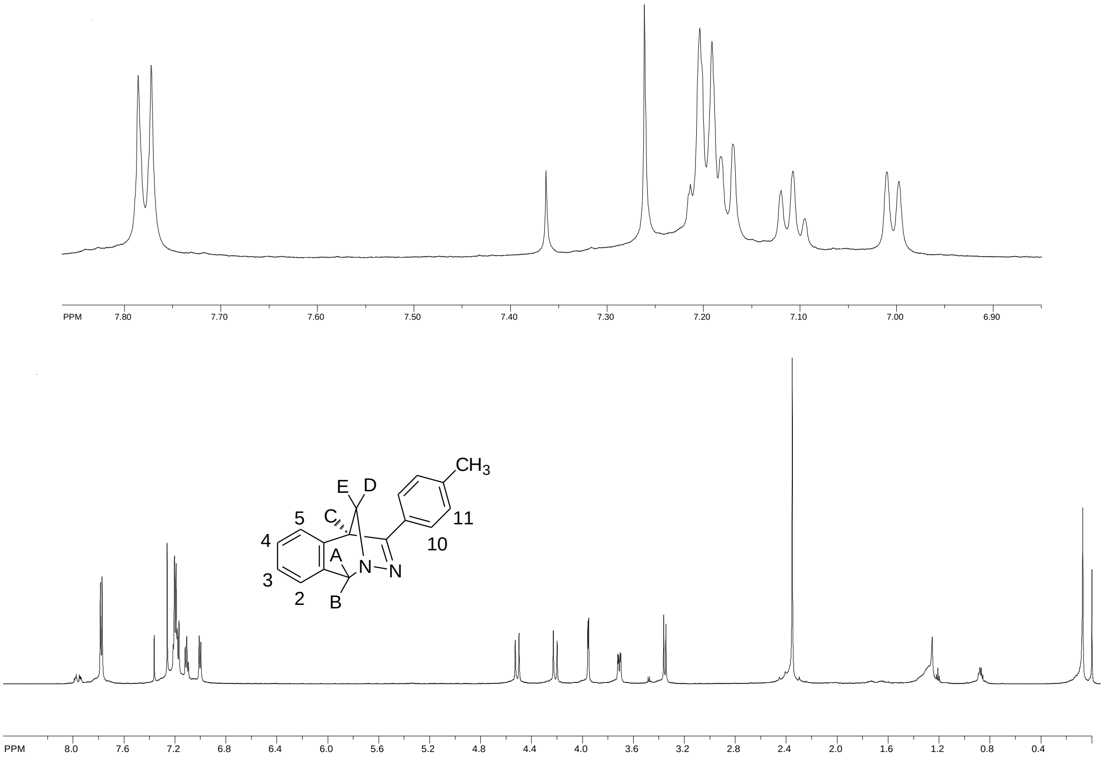

Figure 13: ^{1}H NMR spectrum (600 MHz, $CDCl_3$) of 11-(4-methylphenyl)-9,10-diazatricyclo[7.2.1.0^{2,7}]dodeca-2,4,6,10-tetraene (**15**).

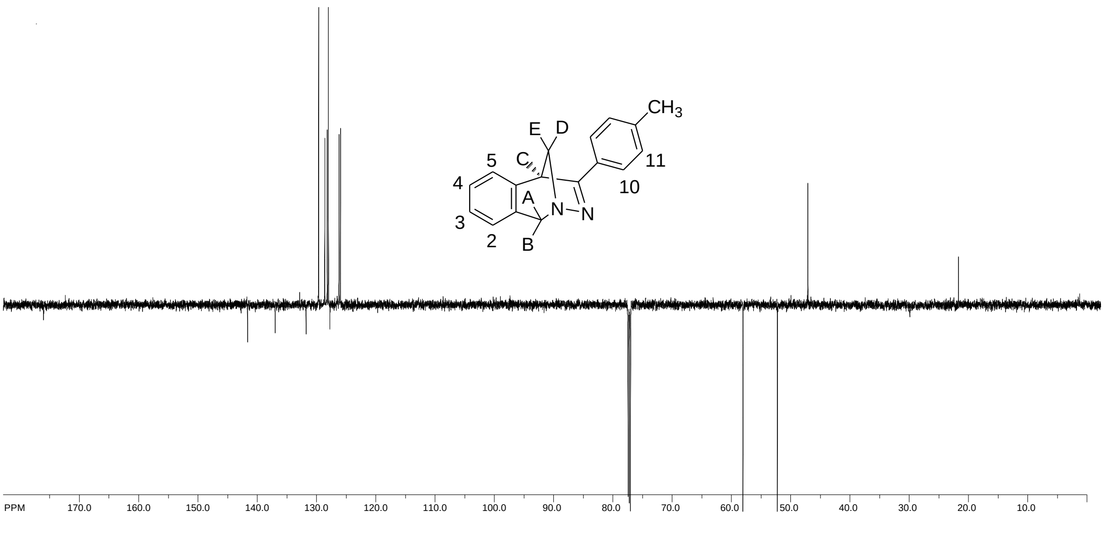

Figure 14: APT spectrum (150 MHz, $CDCl_3$) of 11-(4-methylphenyl)-9,10-diazatricyclo[7.2.1.0^{2,7}]dodeca-2,4,6,10-tetraene (**15**).

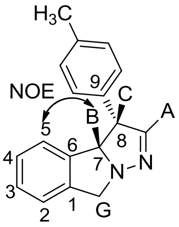

11

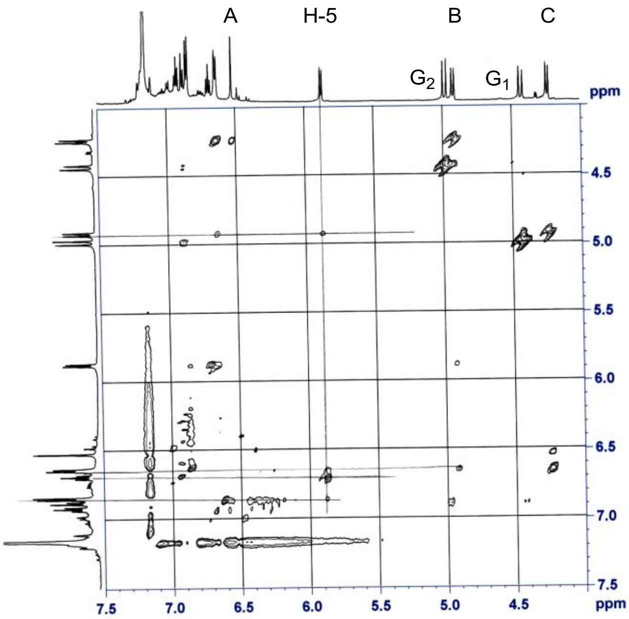

Figure 15: NOESY spectrum of **11**.

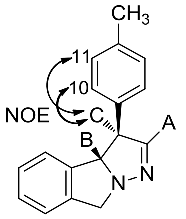

12

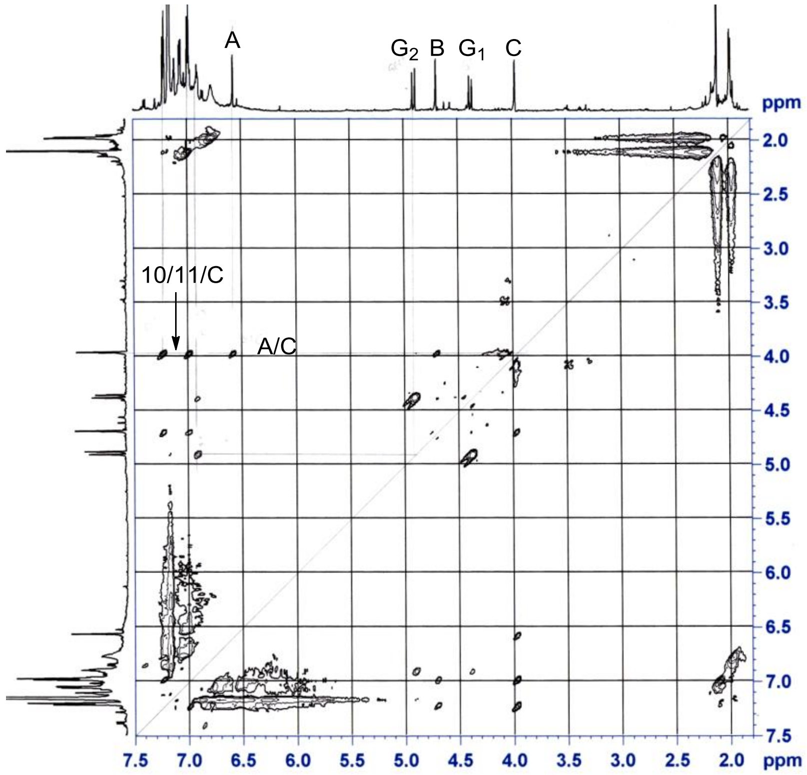

Figure 16: NOESY spectrum of **12**.

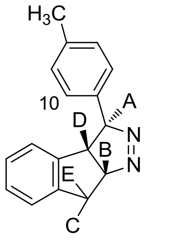

14

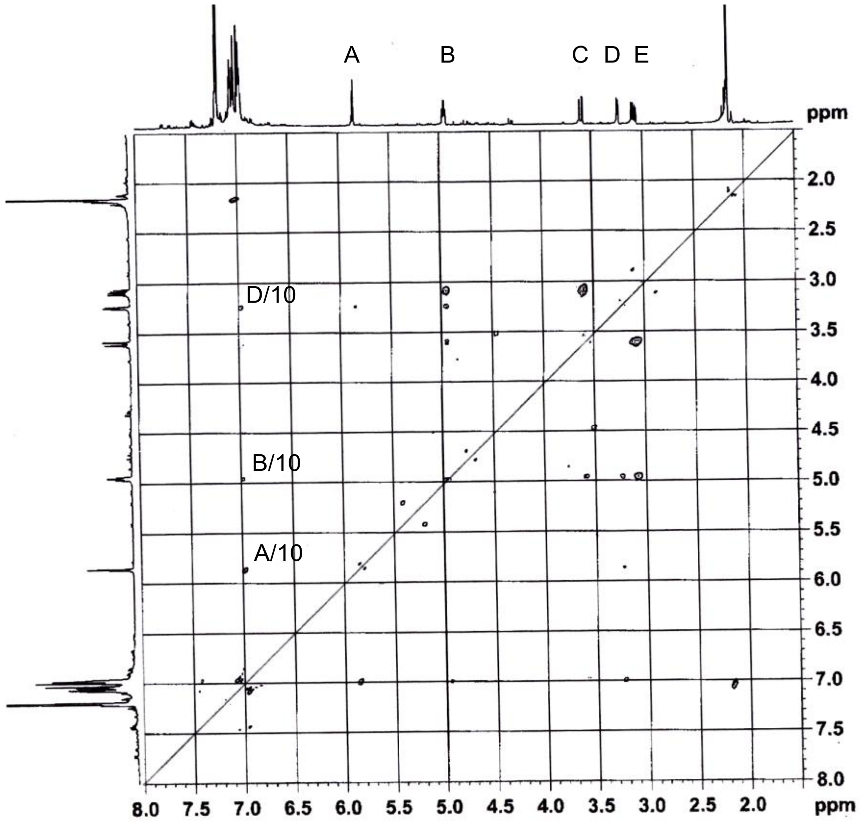

Figure 17: NOESY spectrum of **14**.

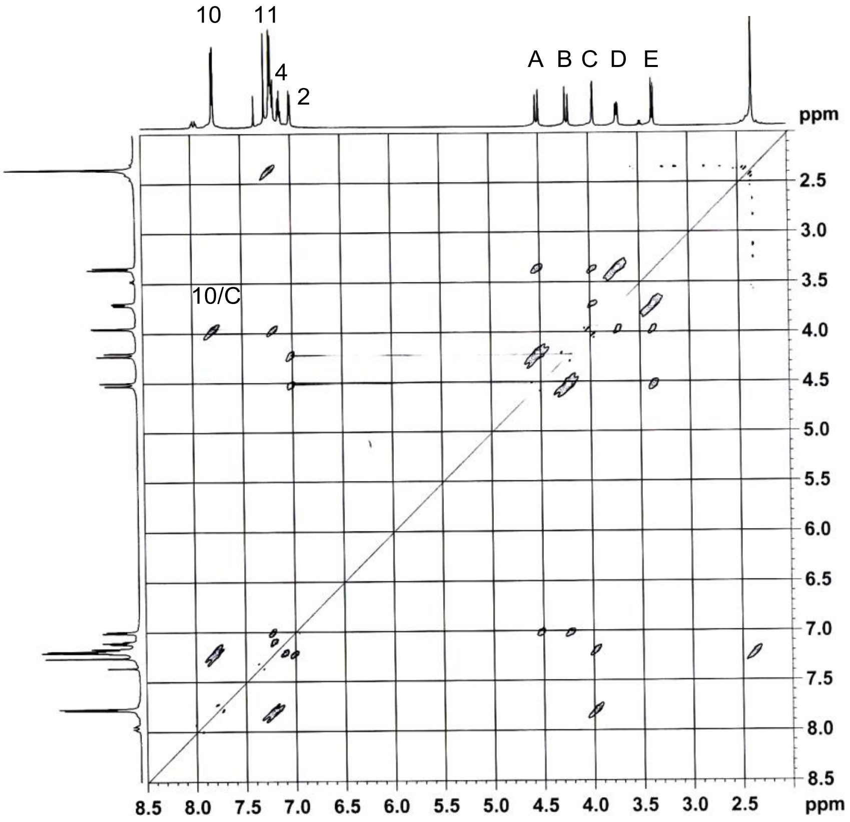

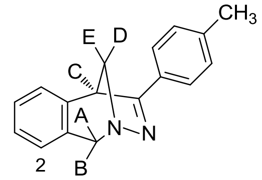

15

Figure 18: NOESY spectrum of **15**.

X-Ray data

Data collection for compound **14** was performed on an Enraf-Nonius CAD-4 diffractometer, with graphite monochromated Cu Kα (1.54179 Å) radiation at room temperature [293(2) K]. The WinGX standard procedure was applied for data reduction [1]. Three standard reflections were measured every 120 minutes as intensity control. Due to the small size of the crystal and the fact that it contains only light atoms, no absorption correction was applied. The structures were solved with SHELXS97 [2] and refined with SHELXL97 [2]. The model was refined by using the full-matrix least-squares refinement against F^2. Hydrogen atoms were refined as riding entities. The atomic scattering factors were those included in SHELXL97 [2]. Molecular geometry calculations were performed by PLATON [3], and molecular graphics were prepared by using ORTEP-3 [4]. Crystallographic, data collection and refinement details are shown in Table S1.

Supplementary crystallographic data for this paper can be obtained free of charge at www.ccdc.cam.ac.uk/conts/retrieving.html (or from the Cambridge Crystallographic Data Centre, 12, Union Road, Cambridge CB2 1EZ, UK; fax: +44 1223 336033; or deposit@ccdc.cam.ac.uk). CCDC 832931 contains the supplementary crystallographic data for this paper.

Table S1: Crystallographic, data collection and refinement details.

	**14**
Empirical formula	$C_{17}H_{16}N_2$
Molar mass / g mol^{-1}	248.32
Crystal size / mm	0.15 × 0.15 × 0.10
Crystal system	Orthorhombic
Space group	$Pca2_1$
a / Å	24.222(1)

b / Å	5.0021(3)
c / Å	11.0572(8)
V / Å^3	1339.70(14)
Z	4
ρ_{calc} / g cm^{-3}	1.231
μ / mm^{-1}	0.563
Θ range / °	3.65–76.22
T / K	293(2)
Range of h, k, l	$0 < h < 30$;<br>$0 < k < 6$;<br>$0 < l < 13$
No. of reflections	1485
Independent reflections	1485
Observed reflections ($I \geq 2\sigma$)	895
R_{int}	0
R (F^2)	0.0466
R_w (F^2)	0.1194
S (Goodness of fit)	1.021
No. of parameters	174
No. of restraints	1
$\Delta\rho_{max}$, $\Delta\rho_{min}$ (eÅ^{-3})	0.125; -0.111

References

(1) Harms, K.; Wocadlo, S. *XCAD-4*, *Program for Processing CAD4 Diffractometer Data*, University of Marburg, Germany, 1995.

(2) Sheldrick, G. M. *Acta Crystallogr.*, *Sect. A*, **2008**, *64*, 112-122.

(3) Spek, A. L. *J. Appl. Cryst.*, **2003**, *36*, 7-13.

(4) Farrugia, L. J. *J. Appl. Cryst.*, **1997**, *30*, 565.
